# Thiamin (vitamin B1, thiamine) transfer in the aquatic food web from lower to higher trophic levels

**DOI:** 10.1371/journal.pone.0308844

**Published:** 2024-12-02

**Authors:** Samuel Hylander, Hanna Farnelid, Emil Fridolfsson, Marc M. Hauber, Vittoria Todisco, Maciej J. Ejsmond, Elin Lindehoff

**Affiliations:** 1 Department of Biology and Environmental Science, Centre for Ecology and Evolution in Microbial Model Systems (EEMiS), Linnaeus University, Kalmar, Sweden; 2 Institute of Environmental Science, Faculty of Biology, Jagiellonian University, Cracow, Poland; University of Connecticut, UNITED STATES OF AMERICA

## Abstract

Micronutrients such as vitamins are transferred from lower to higher trophic levels, but no general ecological concept describes the factors regulating this process. Here, we investigated thiamin (thiamine, vitamin B_1_), which is an example of a metabolically important water-soluble micronutrient. Thiamin is produced by organisms such as bacteria and phytoplankton, and all consumers, such as zooplankton and fish, rely on a continuous intake of thiamin through their diet and possibly from *de novo*-synthesized thiamin by gut microbiota. A deficiency in thiamin negatively affects reproduction in fish and bird populations worldwide. The aim of this study was to quantify thiamin transfer in a planktonic food web in response to thiamin and/or nutrient addition, using an outdoor mesocosm system (an approximately 1.9 m^3^ bag submerged in sea water). These estimates were then compared with literature data on thiamin concentrations at different trophic levels. The results showed that thiamin was rapidly taken up by phytoplankton in both the ambient and nutrient-amended treatments. However, large differences in thiamin concentrations in phytoplankton did not lead to any significant changes in community composition or abundance. Nitrogen addition led to changes in the abundance and community composition of picoplankton and phytoplankton but there were no additional major effects of thiamin addition. Differences in thiamin concentrations in phytoplankton were not detected at the next trophic level in zooplankton. Although the concentrations did not change, a greater abundance of some zooplankton taxa were developed in the thiamin treatments. Comparing the mesocosm results with literature data demonstrated a gradual reduction in thiamin concentrations along the food chain, with six percent of the concentration in producers occurring in top consumers (i.e., piscivorous fish). Overall, these observations illustrate the concept of trophic dilution of micronutrients where concentrations decrease along the food web from phytoplankton via zooplankton and planktivorous fish to piscivorous fish.

## Introduction

A cornerstone of aquatic food web ecology is the understanding of the transfer of macronutrients, such as carbon, nitrogen and phosphorous, from producers to consumers. For example, the potential number of trophic levels in a system is thought to depend on the input of macronutrients, but the actual food web structure is governed by a combination of productivity, ecological stoichiometry and trophic interactions [1–5]. While the dynamics and competition for macronutrients (e.g., nitrogen and phosphorus) are relatively well characterized (e.g., [6–8]), there is little knowledge on the flow and competition for micronutrients such as vitamins, from microbial producers such as bacteria and phytoplankton to consumers such as zooplankton and then to top consumers such as planktivorous and piscivorous fish [9, 10]. The current paradigm is that macronutrients, i.e., nitrogen and phosphorous (sometimes silica), and the relative proportions of these macronutrients (stoichiometry) control organismal growth in most aquatic food webs [5, 7, 11]. Some micronutrients, including iron, are limiting or colimiting in a few systems [11, 12]. It is increasingly thought that vitamins, in some cases, are limiting and potentially structuring various food web interactions [9, 10, 13–16].

One important example of such a micronutrient is thiamin, also known as vitamin B_1_ or thiamine. It is an essential substance in all organisms and is involved in several central cellular processes. Its active form, called thiamin diphosphate, is required for the activity of several enzymes, including pyruvate dehydrogenase, which contributes to the production of acetyl-CoA (for review, see [9]). Thiamin is also a co-factor for transketolase, which is a central metabolic enzyme in both the pentose phosphate pathway and the Calvin cycle [9]. Thiamin loss is large during consumption given its water-soluble nature (e.g., during sloppy feeding), requiring a nearly constant supply of thiamin [17–19]. Thiamin deficiency causes a wide range of health issues in humans and wildlife [18, 20–23]. For example, thiamin deficiency has been observed worldwide in several bird and fish species (e.g., in the. Pacific Ocean, North American Great Lakes, and the Baltic Sea) [20–25]. This phenomenon has been identified as a threat to global biodiversity, potentially leading to population declines [26].

The main thiamin supply in consumers is via diet, as exemplified by the increase in thiamin deficiency in humans whose diet consists mainly of polished rice [18]. The other potential supply of the vitamin is via the gut microbiome. This route is relevant in herbivores with large gastrointestinal tracts, such as ruminants [27, 28], and has also been demonstrated in *Drosophila* [29], but this route is less likely in carnivores such as piscivorous fish, in which the digestive tracts in relation to body size and the volume available for microbiota action are much smaller [30, 31].

Thiamin in aquatic systems is mainly produced by bacteria and phytoplankton, and both groups contain both synthesizers and other taxa that rely partly or entirely on uptake [10, 32–39]. Thiamin supplementation has recently been shown to increase bacterial production, and the inability to produce endogenous thiamin, i.e., thiamin auxotrophy (full or partial), in bacterioplankton appears to be prevalent in metagenomes from widely distributed marine, freshwater and brackish sites [40]. It is also well known that thiamin concentrations in phytoplankton are species specific [41, 42]. For example, thiamin concentrations in filamentous cyanobacteria often surpass those in some green algae, diatoms, dinoflagellates, and cryptophytes [41, 42]. The abiotic environment also affects the concentrations. Abiotic shifts in light, salinity, and temperature have been shown to affect carbon-specific thiamin concentrations in phytoplankton [42]. A main question is therefore whether the community composition of the microbial food web, as well as the amount of available dissolved thiamin, affects the transfer of thiamin from lower to higher trophic levels in the pelagic realm. Current estimates of dissolved thiamin in marine waters are scarce but suggest concentrations in the pM range [43, 44], indicating high competition for this dissolved pool. Concentrations of thiamin in phytoplankton (or more broadly described as seston) are highest during summer and lowest in winter [45]. Thus, it is likely that the availability of dissolved and particulate-bound (in biota) thiamin has direct consequences for the transfer of this organic micronutrient to the food web, but this hypothesis has not yet been thoroughly explored.

Thiamin is then assumed to be transferred via the diet in the pelagic food web to zooplankton and then to fish at higher trophic levels [46, 47]. The potential role of the gut microbiota in providing thiamin to zooplankton and fish is unknown. Considering the dietary transfer pathway via the food web it is expected that pelagic microbial abundance and community composition can regulate thiamin availability and transfer efficiency in the food web [45, 46]. This phenomenon is likely because all consumers such as zooplankton and fish are dependent on thiamin from microbial producers. Recent modeling studies have suggested that food web structure is important for the flow of thiamin from lower to higher trophic levels [46], where thiamin can be funneled via the traditional pathway from microphytoplankton to zooplankton and then to fish or via the microbial loop. These latter food webs are longer and less efficient when micronutrients are transferred from pico-sized producers to flagellates and ciliates to zooplankton than when micronutrients are transferred from microphytoplankton directly to zooplankton. Hence, a shift in the food web structure from shorter to longer thiamin transfer pathways could play a key role in regulating the overall transfer of thiamin in the food web from lower to higher trophic levels.

Here, we studied plankton (bacterio-, phyto- and zooplankton) using a large-scale outdoor mesocosm experiment (approximately 1.9 m^3^ bags submerged in the sea) designed to explore whether nitrogen and thiamin addition, or combinations of the two, affect community composition and vitamin concentrations in primary producers such as phytoplankton and in consumers such as zooplankton. Nitrogen is often the limiting nutrient for production in this system (Baltic Sea, [48]) and we hypothesized that a pulse of nitrogen, individually or combined with thiamin (e.g,. during vertical mixing), would shift the phytoplankton community structure and the trophic transfer of thiamin to zooplankton. Thiamin deficiency has been observed in top predators in the Baltic Sea for decades [49–52] (the location of this study), meaning that background information on thiamin concentrations in the Baltic Sea food web is available [45–47]. Together with data from other systems, we then compared our results of thiamin concentrations in phytoplankton and zooplankton with data collected from the literature, quantifying the concentrations of thiamin in the food web from producers to top consumers (i.e,. phytoplankton, zooplankton, planktivorous fish, and piscivorous fish). We hypothesized that concentrations decrease from producers to top-consumers, and this work is the first large-scale study of thiamin availability in the food web from primary producers to consumers, thus providing critical insights into the role of micronutrients in food web ecology.

## Methods

### Mesocosm experimental setup

A factorial design was used to investigate how thiamin and nitrogen supplementation affect the transfer of thiamin from microbial food webs to zooplankton (S1 Fig S5 in S1 File). The experiment was performed outdoors close to the Tvärminne Zoological Station, Finland, using a floating rectangular-shaped mesocosm platform located in open water near the coast (59° 50,591`N 23° 15,680`E), with a total depth of 8 m. Fourteen mesocosm plastic bags 3 m in length and 1 m in diameter were attached to one long side in an east-to-west direction (S5 Fig in S1 File). Each bag was covered with Plexiglas covering the entire opening and secured approximately 1 m above the bag opening to allow gas exchange. All the bags were put in place in the morning of 31-05-2019 within 3 h, starting simultaneously from both ends of the platform. The folded bags were lowered to approximately 1.5 m depth and allowed to unfold and draw in surrounding Baltic Sea water, including a full plankton community, through a mesh (2 mm mesh size) covering the opening of the bags aimed at excluding small macroinvertebrates and fish. The experiment started the following day (01-06-2019) and ended after 16 days (17-06-2019). The outermost bag on each side was not included in the study but was left in place to ensure the same light and turbulence conditions for all bags, resulting in a total of 12 experimental units. The total volumes of the mesocosm bags were measured at the finalization of the project by the addition of NaCl and the measurement of changes in mS cm^-1^. The final volume of the bags was 1.85±0.2 m^3^. Approximately 60 L was sampled from each bag during the experiment, providing an estimation of the initial volume to be 1.92±0.2 m^3^.

Nitrogen, in the form of nitrate (NO_3_^-^) and/or thiamin, in the form of free thiamin (TF, C_12_H_17_N_4_OS^+^), was added once at the beginning of the experiment to mimic a pulse of macro- and micronutrients (e.g., after a mixing event) in a triplicate factorial design with the following treatments: **C**: low thiamin-low nitrogen; **N**: low thiamin-high nitrogen; **Thi**: high thiamin-low nitrogen; and **N+Thi**: high thiamin-high nitrogen. Dissolved nutrients were sampled at the location three days prior to the experiment, and from those results, NaNO_3_ was added to treatments N and N+Thi for a total nitrogen (NO_3_^-^, NO_2_^-^, NH_4_^+^) of 4 μM. Phosphate was adjusted to 0.5 μM using NaH_2_PO_4_ * 2H_2_O in all treatments, aiming for an N:P ratio of 8 in the N and N+Thi treatments and 4 in treatments C and Thi. This ratio and absolute concentration of nitrogen and phosphorus in treatments N and N+Thi correspond to conditions in the Baltic Sea. Thiamin was added to reach a concentration of 296.5 nM in the mesocosms (excluding the dissolved thiamin, which may already be naturally present). Similar concentrations of dissolved thiamin are used in phytoplankton growth media to produce nonlimiting conditions and good phytoplankton growth [53]. This was done by mixing free thiamin (Thiamin hydrochloride, SIGMA Life Science) in deionized water with a one liter stock solution. Smaller subsamples of the stock solution were added to each mesocosm to reach the target concentration. Estimates of dissolved thiamin concentrations in aquatic systems are scarce but tend to range from nondetectable to approximately 350 pM [13, 43, 44, 54, 55]. Hence, the applied concentration is higher than that found in the available field estimates but was applied to ensure nonlimiting conditions of dissolved thiamin. Furthermore, high concentrations were applied since this molecule is easily degraded and removed from the system in response to changes in environmental factors such as temperature and light exposure [56, 57].

### Sampling of water and organisms

Each mesocosm bag was mixed with a Secci disk on a rope before sampling of 10 L (15 L when zooplankton were included in the sampling) at 2 m depth using a water collector (Limnos). Daily measurements of pH, temperature and salinity were performed using a multiparameter meter (VWR MU 6100 H).

### Nutrients

Samples for nutrient analysis (50 mL) were collected on Days 0, 3, 6, 9, 12 and 16 and frozen (-20°C) until analysis. The concentrations of ammonium (NH_4_^+^), nitrate and nitrite (NO_3_^-^ + NO_2_^-^), phosphate (PO_4_^3-^) and silica (SiO_2_) were measured with an automated analyzer (Aquakem 250).

### Chlorophyll as a proxy of phytoplankton biomass

Samples for chlorophyll *a* (Chl *a*) were taken daily by filtering 100 mL of seawater onto A/E glass fiber filters (Sterlitech Corporation, United States, pore size approximately 1 μm), which were stored at -20°C until analysis. Chl *a* was extracted using 10 mL of ethanol (98%) overnight [58], and fluorescence (ex 430:em 670 nm) was measured using a Varian Cary eclipse Fluorescence spectrophotometer (Agilent Technologies, United States). The Chl *a* concentration (μg L^-1^) of the samples was determined by comparison to a calibration curve of known concentrations.

### Flow cytometry to quantify picoplankton

Flow cytometry samples were collected daily in duplicate 2 ml cryovials and fixed at a 2% final concentration with EM-grade glutaraldehyde. The tubes were mixed by inversion and incubated at room temperature for 5 min prior to storage at -80°C until analysis. Autotrophic picoplankton and heterotrophic bacterial cells were counted in technical duplicates using a Cube8 flow cytometer (Sysmex Partec GmbH, Germany). Samples for bacterial abundance were stained with SYBR Green (Life Technologies). Autotrophic picoplankton were identified based on forward scatter as a proxy for cell size and the red fluorescence signal from the 488 nm laser as a proxy for Chl *a* content.

### DNA sampling and extraction

Nucleic acid samples were collected on Days 0, 3, 6, 10, 12 and 16 by gentle vacuum filtration of 200 ml of sample through a 47 mm, 0.2 μm Supor membrane filter (Pall Corporation). The filters were placed in 2 ml cryovials and immediately frozen at -80°C until further processing. DNA was extracted using the FastDNA® SPIN Kit for soil (MP Biomedicals) with Matrix E beads according to the manufacturer’s protocol with a few modifications (see supplementary material).

### Characterization of the composition of prokaryotic and eukaryotic plankton communities by PCR amplification and sequencing of 16S and 18S rRNA gene fragments

For microbial community composition analysis, 16S and 18S rRNA gene fragments were amplified using a two-step PCR protocol and sequenced using Illumina MiSeq (2 x 300 bp). All PCRs were performed using Phusion High-Fidelity PCR (New England Biolabs Inc., United States) Master Mix (Thermo Scientific). Each reaction was prepared in duplicate, and negative controls containing only ultrapure water were included. In the first step, primers specific to the gene targets with overhang adaptors were used. For the 16S rRNA gene fragment, 341F (CCTACGGGNGGCWGCAG) and 805R (GACTACHVGGGTATCTAATCC) primers amplifying the V3-V4 region were used (Herlemann et al. 2011; Hugerth et al. 2014) [59, 60] and for the 18S rRNA gene fragment, 574*F (CGGTAAYTCCAGCTCYV) and 1132R (CCGTCAATTHCTTYAART) (Hu et al. 2016) were used at a concentration of 0.5 μM. Sample-specific indices and adaptors for Illumina sequencing were added in a subsequent PCR of 12 cycles of Nextera DNA Dual-index (N7-N5). Following each PCR, PCR amplicons were purified using the Agencourt AMPure XP PCR purification kit (Beckman Coulter). The samples were subsequently sequenced at the National Genomics Infrastructure (NGI) Sweden SNP&SEQ Technology Platform at Uppsala University, Sweden. The amplicons are available in the Sequence Read Archive. The sequences are available in GenBank under the accession numbers OQ644547-OQ644563

### Bioinformatic processing

The raw sequence data were demultiplexed at the facility and quality controlled using the nf-core/ampliseq (10.5281/zenodo.1493841v1.2.0; [61]) pipeline, which was run on Nextflow v.10.20.0. Amplicon sequence variants (ASVs) were inferred using DADA2 [62] in QIIME2 2019.10 [63]. The SILVA v132 database was used for classifying ASVs for the 16S rRNA gene amplicon data [64]. As the amplicon lengths of the 18S rRNA fragments were too long to allow for merging of the forward and reverse sequences, the classification was performed using the forward read only [65], using GenBank BLASTN against the PR2 database version 4.14.1 [66].

### Composition of phytoplankton and zooplankton communities

Samples for phytoplankton identification and enumeration were collected on Days 0, 3, 6, and 16 by fixing 50 mL sample with acid Lugol’s solution and were stored in the dark until analysis via inverted light microscopy. The dominant phytoplankton and ciliates were identified to species or genus, and the biovolume (μm^3^) and biomass (μg C) were calculated as previously described [67, 68]. Samples for zooplankton identification and enumeration were collected on Days 0, 6 and 16. Five liters from each mesocosm was filtered through an 80 μm net, resuspended in 50 ml of filtered seawater and fixed using acid Lugol’s solution. Zooplankton were identified to the genus or species level when possible, using a stereomicroscope (Olympus SX7). Species were also genetically identified for individual specimens that were picked under a stereomicroscope, washed three times in sterile filtered seawater, placed in 1.5 ml Eppendorf tubes and stored at -80°C. Cells were lysed and amplified using Phire Tissue Direct PCR Master Mix (Thermo Scientific). Briefly, 20 μl of dilution buffer and 0.5 μl of DNARelease Additive were added to each tube and incubated at room temperature for 5 min, followed by incubation at 98°C for 2 min. Then, 1.5 μl of the solution was used as a template for PCR. The gene target used for identification was mitochondrial cytochrome oxidase I (mtCOI), which was amplified with the forward primer LCO-1490 and reverse primer 1 HCO-Co-23585 or reverse primer 2 HCO-21985 [69] at a concentration of 0.5 μM each with an annealing temperature of 48°C. The samples were sent to Macrogen Europe B.V. for purification and Sanger sequencing. The closest relatives were identified via BLASTN in GenBank.

### Measurements of thiamin and particulate carbon

Samples for particulate thiamin and particulate carbon content analysis were collected from total water and from five size fractions, >200 μm, 200–90 μm, 90–20 μm, 20–3 μm and 3–0.7 μm, on Days 0, 6 and 16. The seawater (9 L) was subsequently filtered, and samples from the different size fractions and 50 ml samples were collected on Whatman GF/F filters (25 mm), except for the 20–3 μm fraction that was collected on GF/D filters (25 mm,) and 1,000 ml was filtered. For the smallest size fraction, 3–0.7 μm 1000 ml of filtrate from the GF/D filter was collected on a GF/F filter. Samples for thiamin analyses were stored in Eppendorf tubes at -80°C until further analysis. Samples for particulate carbon content analysis were collected on precombusted (475°C, 2 h) filters and stored in H_2_O_2_-washed Eppendorf tubes at -20°C until further analysis. The particulate carbon content was analyzed using a Perkin Elmer CHNS/O Analyzer 2400 Series II (Perkin Elmer United States). Prior to analysis, the samples were dried at 60°C for at least 24 h and stored in a desiccator.

Thiamin was analyzed as previously described [70] with minor modifications consistent with other reports [42, 47]. Briefly, thawed samples were sonicated in 0.1 M HCl (1–1.5 mL) with a Vibra-Cell sonicator on ice (amplitude: 92%; pulse: 1 s; duration: 1.5 min). Subsequently, the samples were centrifuged at 16,900 × g for 10 min at 10°C, after which 700 μL of the supernatant was centrifuged once more under the same conditions. Next, 600 μL of the supernatant was mixed with 550 μL of MeOH, 300 μL of 1 M NaOH and 50 μL of freshly made 30 mM K_3_Fe(CN)_6_. The mixture was then filtered through a 0.45-μm PTFE/PP syringe filter. Blanks consisting of 0.1 M HCl and standard solutions (1 μM) of three types of thiamin, free thiamin (TF), thiamin monophosphate (TMP) and thiamin diphosphate (TDP), were prepared in 0.1 M HCl and aliquoted in a five-point standard series. Blanks and standards were treated similarly to the samples, except for sonication and centrifugation.

Thiamin samples were analyzed using a Hitachi Chromaster HPLC system (Hitachi, Japan) with a ReproSil-Pur 120 NH2 column (5 μm particle size, 4.6 mm [I.D.]×250 mm) protected by a ReproSil-Pur 120 NH2 guard column (5 μm particle size, 4 mm [I.D.]×4 mm) and a fluorescence detector (excitation wavelength: 375 nm; emission wavelength: 450 nm). The samples were kept in the autosampler at 4°C, and the column oven was set to 30°C. A volume of 100 μL was injected, and the samples were analyzed at a flow rate of 1 mL min–1. The mobile phase consisted of MeOH:0.1 M phosphate buffer (pH 7.4) under isocratic conditions (43:57). Chromatograms were integrated using the software OpenLab (Agilent Technologies), and baselines were drawn automatically and later manually inspected for quality checking. Three types of thiamin were analyzed, TF, TMP and TDP, and these values were summed to obtain the total thiamin content (Ttot).

### Literature survey on thiamin concentrations at different trophic levels

To better understand the mesocosm results, published estimates of thiamin concentrations in field-sampled phytoplankton (or seston), zooplankton, planktivorous fish, piscivorous fish and omnivorous fish were collected from the literature. Phytoplankton and zooplankton data were collected using the search strings “thiamin” OR “thiamine” OR “vitamin B1 AND “seston/phytoplankton/zooplankton,” whereas fish data were collected using the search strings “thiamin” OR “thiamine” AND “fish” within certain Web of Science categories (Fisheries OR Marine Freshwater Biology OR Veterinary Sciences OR Environmental Sciences OR Limnology OR Biology OR Physiology OR Oceanography OR Zoology). The first searches yielded 274 references for which the titles and abstracts were assessed. Data were only included if they were sampled in the field (no experimental or bag data), quantified via high-performance liquid chromatography (HPLC, only references from 1998 or later since new methods were developed at that time [71]), and if the amount of thiamin in nmol/g was normalized for weight. We omitted estimates if they were normalized per individual or per liter. For fish, we only included data presenting thiamin as nmol per wet weight in muscle or in whole fish, and for plankton, we included only data normalized per carbon (to avoid too many uncertain conversions). The list of appropriate references was also compared with that of Balk et al. (2016), in which similar data were summarized for some fish, leading to 30 included references in total for all trophic levels [24, 45, 51, 71–97]. If several estimates were available for a species of zooplankton or fish, an average value was calculated. The seston data included phytoplankton of many different species, and the data from each sampling occasion were included since they likely reflect different community compositions. When the phytoplankton data were divided into different size categories, we summed the total thiamin content. Zooplankton data were available either for individual taxa (at the genus level) or for mixtures of total zooplankton (mixed zooplankton samples were regarded as separate species in the analysis). The type of feeding habit of the fish was defined using the description in fishbase.se. Benthophagous fish species were rare and were therefore combined with omnivorous species. All the data were converted to nmol gC^-1^. This is the conventional unit for plankton. Recalculation of the fish data to thiamin per gram of carbon (gC^-1^) was performed assuming a moisture content of 73.6% and a carbon content of 43.6% of the dry weight [98].

### Data handling, statistics and permits

All data handling, statistical analyses and graphics were created using R, version 3.6.0 [99] and Adobe Illustrator. For linear mixed effects analyses (LMMs) [100, 101] (lme4, car packages), the mesocosm was specified as a random effect, and the results are presented as chi square (χ2) values and P values in addition to subsequent post hoc tests, which are presented as z or t values. When samplings were performed more than three times during the mesocosm study, we assessed significant differences (nitrogen addition, thiamin addition, and their interactions) using a mixed model with generalized least squares (GLS) methods and a compound symmetry correlation structure to account for potential autocorrelation (nlme, car packages). Nonmetric multidimensional scaling (NMDS) was performed using the vegan package, and the Bray‒Curtis distance measurement was used. The 16S and 18S rRNA gene amplicon data have been submitted to the Sequence Read Archive (SRA) with BioProject ID PRJNA1051975, and all other data are available in the supplementary material. No particular permits were needed to conduct this study.

## Results

### Abiotic conditions in the mesocosms

Nutrient supplementation had the intended effects, with generally greater NO_2_-NO_3_ availability in the N and N+Thi treatments than in the C and Thi treatments, but thiamin addition had no measurable effect (S1 Figs S1-2 in S1 File). The addition of nitrate NO_3_^-^ to nitrogen treatments increased the concentrations of nitrite+nitrate NO_2_^-^+NO_3_^-^ on Day 0 in treatments N (3.5±0.6 μM) and N+Thi (3.7±0.7 μM) compared to those in treatments C (0.1±0 μM) and Thi (0.2±0.1 μM). At the next sampling, on Day 3 and until Day 16, all treatments had NO_2_^-^+NO_3_^-^ concentrations <0.1 μM. These results indicate that there was an interactive effect between nitrogen addition and time on NO_2_^-^+NO_3_^-^ concentrations but no effect of thiamin addition (GLS, thiamin addition: χ^2^ = 0.05, p = 0.8; nitrogen addition: χ^2^ = 135.0, p<0.01; time χ^2^ = 20.3, p = <0.01; N:time: χ^2^ = 20.3, p<0.01; no other interactive effects p>0.05). The initial addition of PO_4_^3-^ resulted in a concentration of 0.3 μM on Day 0 in all the treatments. On Day 3, the PO_4_^3-^ concentration decreased to 0.2 μM in the C and Thi treatments and 0.1 μM in the N and N+Thi treatments, and these levels were maintained during the experiment (S1 Fig S2 in S1 File) with lower concentrations in response to N addition and over time but no thiamin effect (GLS, Thiamin addition: χ^2^ = 2.5, p = 0.1; Nitrogen addition: χ^2^ = 58.0, p<0.01; Time χ^2^ = 61, p<0.01.1, no interactive effects p>0.05). Therefore, the nutrient dynamics in the mesocosms are consistent with the intended nitrogen fertilization effect followed by an increase in production (see Chl *a* below). The other measured constituents (SiO_3_, NH_4_^+^, totN, totP and ratios) are reported in the supplementary material. The pH was also greater in the nutrient-amended treatments, likely as a result of greater production (photosynthesis). In the treatments with N addition (N; N+Thi), the pH increased from 8.26±0.02 (all treatments) to 8.45±0.03 (N) and 8.46±0.01 (N+Thi) on Day 4, reaching a maximum on Day 10 at pH values of 8.56±0.02 (N) and 8.53±0.02 (N+Thi) (S1 Fig S1 in S1 File). These results indicate that there was an interactive effect between nitrogen addition and time on pH but no effect of thiamin addition (GLS, thiamin addition: χ^2^ = 0.005, p = 0.9; nitrogen addition: χ^2^ = 386.5, p<0.01; time χ^2^ = 0.2, p = 0.6; N:time: χ^2^ = 30.0, p<0.01; no other interactive effects p>0.05). In treatments C and Thi, the pH remained lower than 8.3 throughout the experiment. The temperature increased during the experiment from 11.18±0.17°C on Day 0 to a maximum of 18.30±0.30°C on Day 7, after which the temperature decreased to 13.67±0.15°C on Day 12 and 14.78±0.19°C on Day 16 (S1 Fig S1 in S1 File). All mesocosm bags followed the same general patterns in temperature, with differences with time and slightly higher temperatures in response to both thiamin and N addition (GLS, thiamin addition: χ^2^ = 10.0, p<0.01; nitrogen addition: χ^2^ = 7.3, p<0.01; time χ^2^ = 66.7, p<0.01, no interactive effects p>0.05). There was an 8°C difference in temperature over time, but the difference among treatments was smaller, on the order of a 0.1–0.4°C difference among treatments. The average temperatures were 14.6 ± 2.3, 14.5 ± 2.2, 14.2 ± 2.2, and 14.4 ± 2.2°C in the N+Thi, Thi, C and N treatments, respectively (average ± SD). The reasons for these small differences in temperature among treatments are not known and are not thought to significantly affect plankton dynamics.

Hence, nutrient addition generally led to greater availability of nitrogen in the N addition treatments, but thiamin addition generally had little effect on the water chemistry variables. Detailed results regarding these variables are available in the supplementary information (S1 Figs S1-2 in S1 File).

### Biomass of phytoplankton (Chl a) and abundance of picoplankton

**Fig 1:**
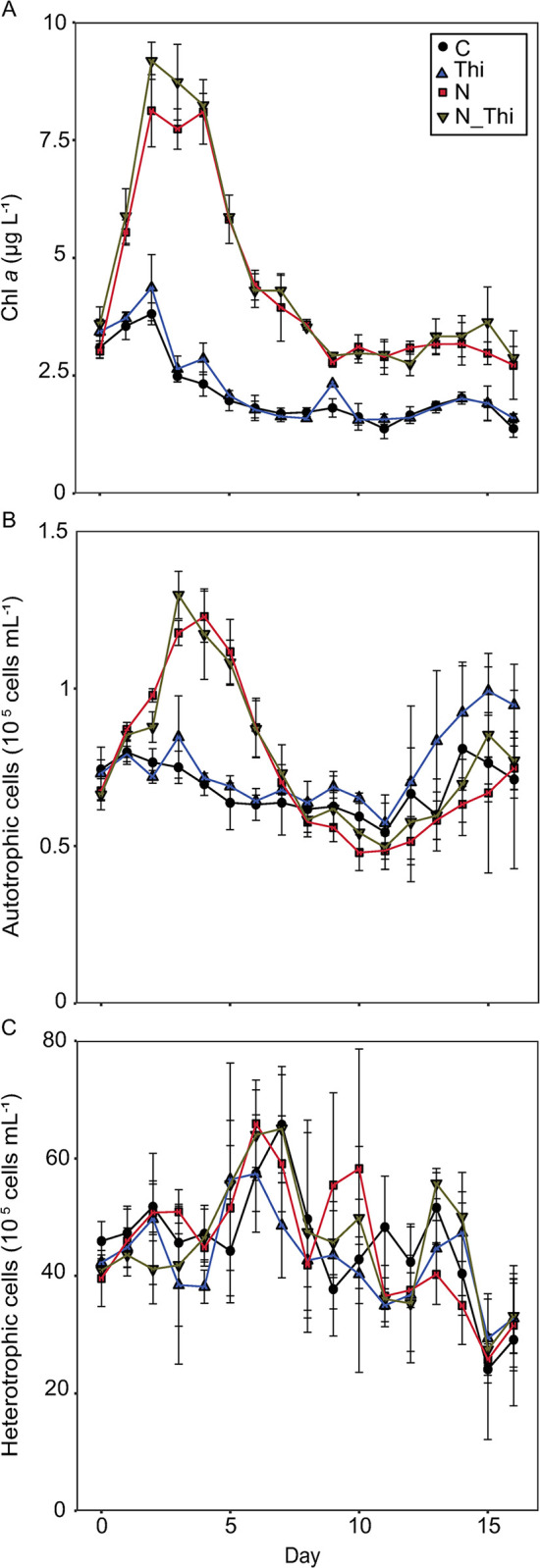
The average chlorophyll *a* (A) and cell abundances of autotrophic (B) and heterotrophic picoplankton (C) were determined via flow cytometry at each time point. Error bars show standard deviations for triplicate treatments: control (C), thiamin (Thi), nitrogen (N), and thiamin + nitrogen (N_Thi). For significant differences in response to treatments, see the text.

The initial Chl *a* concentration did not differ among treatments (3.29±0.33 μg L^-1^). The maximum Chl *a* concentration was reached on Day 2 in all treatments; afterward, the Chl *a* concentration decreased to the initial level (N; N+Thi) or lower (C; Thi) (Fig 1). In the treatments with N added (N; N+Thi,) the Chl *a* increased to 8.12±0.76 μg L^-1^ (N) and 9.18±0.39 μg L^-1^ (N+Thi). The maximum Chl *a* concentrations in treatments C (3.81±0.23 μg L^-1^) and Thi (4.37±0.70 μg L^-1^) were lower than those in treatments with N addition and remained lower throughout the experiment (Fig 1A). These results indicate that there was an interactive effect between nitrogen addition and time on Chl *a* but no effect of thiamin addition (GLS, thiamin addition: χ^2^ = 3.6, p = 0.06; nitrogen addition: χ^2^ = 498.2, p<0.01; time χ^2^ = 135.2, p = <0.01; N:time: χ^2^ = 21.8, p<0.01; no other interactive effects p>0.05).

At the start of the experiment, autotrophic picoplankton responded quickly to the treatments and more than doubled in the N and N+Thi treatments compared to those in the C and Thi treatments (Fig 1). The peak in abundance occurred on Day 3 (average of 1.2 × 10^5^ cells ml^-1^) for the N treatment and on Day 4 (average of 1.3 × 10^5^ cells ml^-1^) for the N+Thi treatment. After the initial bloom, the abundances decreased again and reached levels similar to those in the C and Thi treatments on Day 7. After Day 12, the general trend was that the abundance of autotrophic picoplankton started to increase again, but there was greater variation among the replicate mesocosms (Fig 1B). The concentrations of autotrophic picoplankton were slightly greater in the thiamin treatments during some parts of the experiment, and there were therefore interactive effects between nitrogen addition and time and between thiamin addition and time (GLS, thiamin addition: χ^2^ = 0.8, p = 0.36; nitrogen addition: χ^2^ = 2.3, p = 0.13; time χ^2^ = 17.5, p = <0.01; N:time: χ^2^ = 32.1, p<0.01; Thi:time: χ^2^ = 5.5, p = 0.02; no other interactive effects p>0.05). The heterotrophic picoplankton ranged between 2.4 × 10^6^ and 6.6 × 10^6^ cells ml^-1^ (Fig 1C). Unlike the observations for autotrophic picoplankton, there was no clear response to the different treatments in terms of the abundance of heterotrophic picoplankton (Fig 1; GLS, thiamin addition: χ^2^ = 0.2, p = 0.63; nitrogen addition: χ^2^ = 0.1, p = 0.0.75; time χ^2^ = 212.1, p = <0.01, no interactive effects p>0.05).

### Prokaryotic and eukaryotic plankton community composition based on 16S and 18S rRNA gene amplicon sequencing

**Fig 2:**
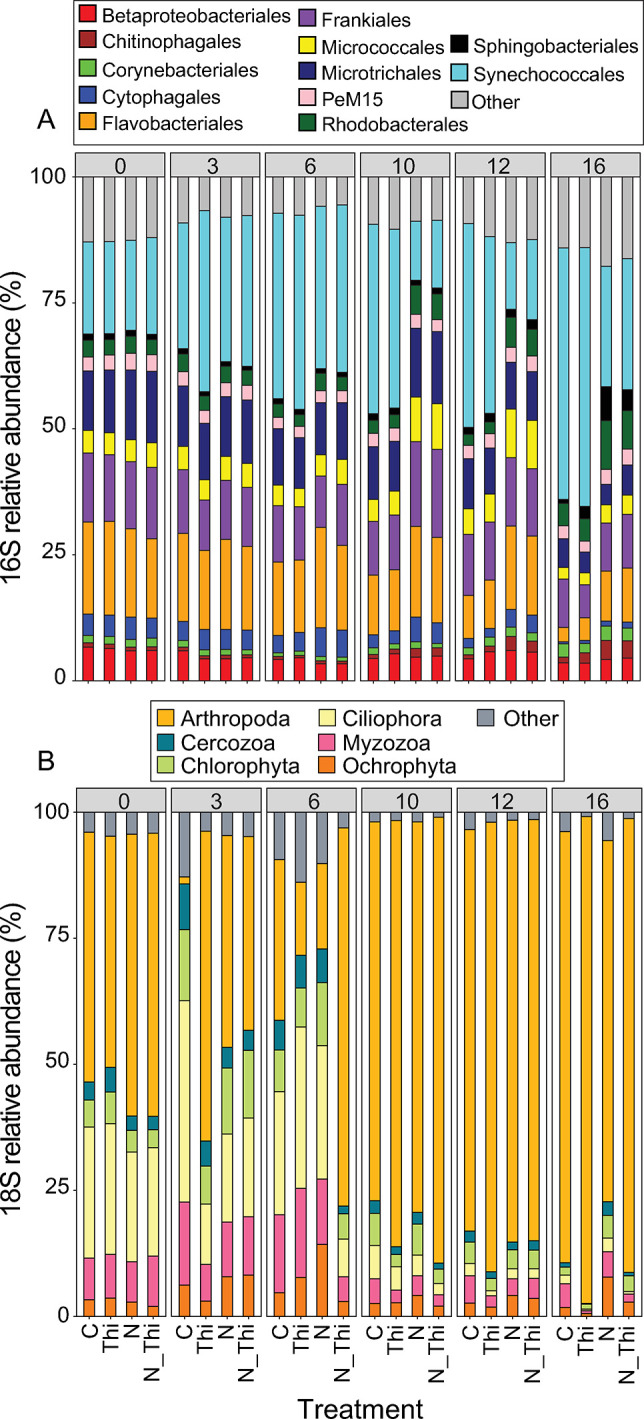
Stacked bar graph of the average relative abundance and the taxonomical identification in the 16S rRNA (A) and 18S rRNA (B) gene amplicon libraries for each time point (Day 0, 3, 6, 10, 12 and 16) for triplicate treatments; Control (C), Thiamin (Thi), Nitrogen (N), and Thiamin + Nitrogen (N_Thi). The remaining ASVs have been grouped in the category “Other”, and ASVs with the same identification have been given indices in the legend.

At the start of the experiment, the bacterial community composition in the bulk size fraction (>0.2 μm) consisted of a diverse community typical of Baltic Sea summers, with *Synechococcales* and *Flavobacteriales* being the orders with the highest relative abundances (Fig 2A). By Day 10, the bacterial community composition in the N and N-Thi treatments was clearly separated from that in the C and Thi treatments, with *Synechococcales* beginning to be associated with the nonnitrogen treatments (S3B Fig in S1 File). However, there was no separation in response to thiamin treatment. The order that fluctuated the most during the duration of the experiment and with the different treatments was *Synechococcales*. The highest proportions of *Synechococcales* were detected in the C and Thi treatments on Day 16, reaching an average of approximately 50% of the sequences in the 16S rRNA gene amplicon libraries (Fig 2A).

The eukaryotic photoautotroph community was investigated using classification of the 340 identified 16S ASVs affiliated with chloroplasts. In total, the chloroplast ASVs represented 0–11% of the sequences in the amplicon libraries (S1 Table S1 in S1 File). Chlorophyta and Haptophyta were the dominant phyla, with 47.8% and 44.8% of the chloroplast sequences, respectively (S1 Fig S4 in S1 File). The communities were generally separated in response to nitrogen addition, whereas the communities in the treatments with and without thiamin were similar (S1 Fig S3A in S1 File). For example, nitrogen addition was associated with Chlamydomonadaceae and *Chaetoceros*, whereas nonnitrogen treatments were associated with, e.g., *Chlorella*.

At the start of the experiment, the eukaryotic community, based on 18S rRNA gene amplicon sequencing, was dominated by reads from Arthropoda and Ciliophora (Fig 2B). ByDay 10, all libraries were dominated by Arthropoda (>75% of reads,) while the proportion of Ciliophora had decreased (Fig 2B and S1 Fig S3B in S1 File). At the end of the experiment, on Day 16, the proportion of Arthropoda was greatest in the thiamin treatments (Thi and N_Thi; Fig 2B). Using this proxy of community composition, there was no distinct separation of communities in response to the treatments (S1 Fig S3C in S1 File).

### Phytoplankton community composition

**Fig 3:**
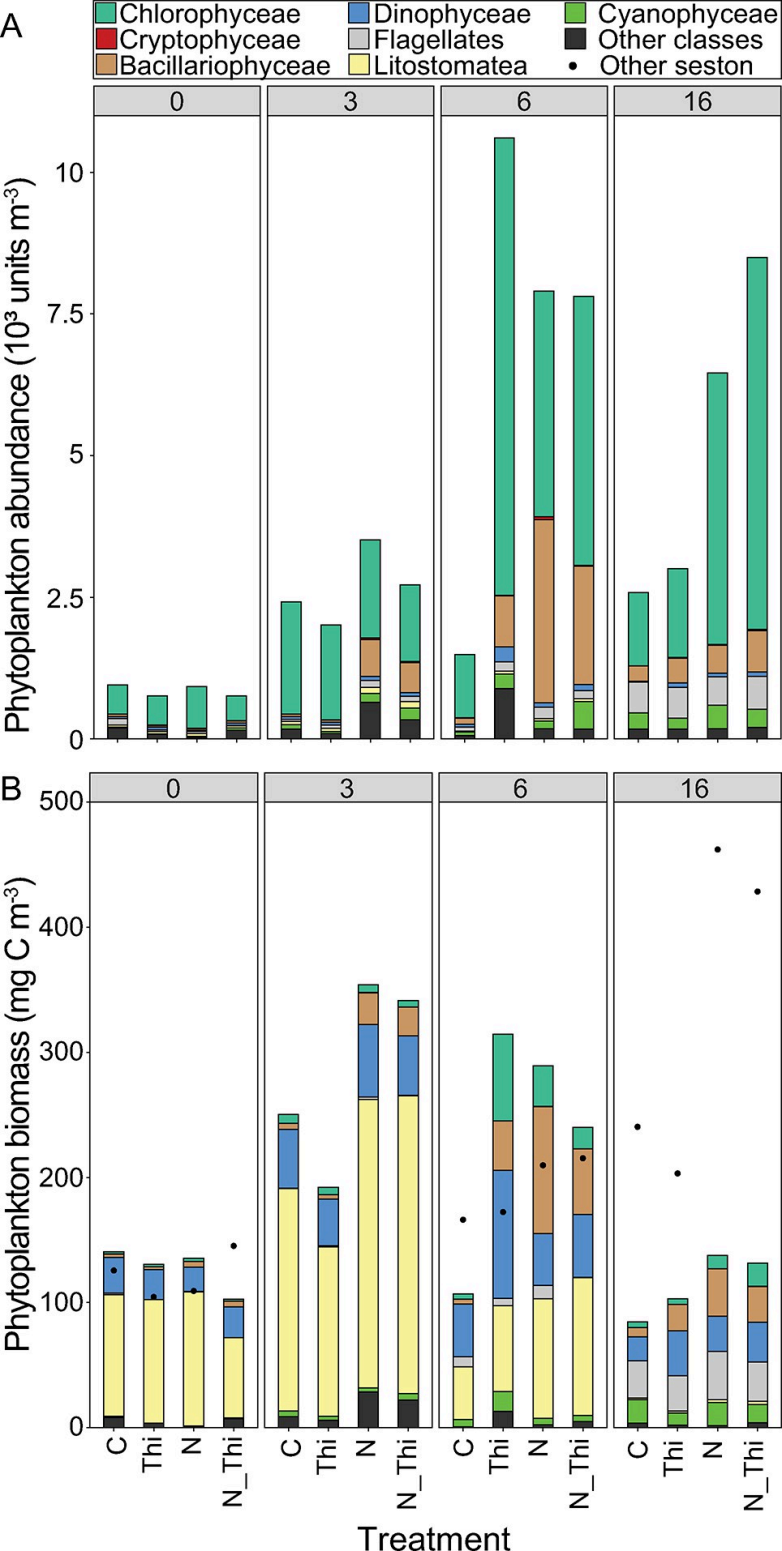
Phytoplankton abundance (10^3^ units m^-3^) (A) and biomass (mg C m^-3^) (B) for the dominant taxonomic groups in the Control (C), Thiamin (Thi), Nitrogen (N), and Thiamin + Nitrogen (N_Thi) treatments. There were overall higher abundances following N addition but no effects of thiamin addition (see statistics in the text). However, there were no significant differences among treatments in either abundance or biomass within each sampling occasion, i.e., on Days 3, 6 or 16 (Mann‒Whitney U test, multiple comparisons, p>0.05)).

Phytoplankton cell abundance (unit m^-3^) was dominated by Chlorophyceae (genus *Monoraphidium*) in all treatments and sampling occasions (Fig 3A). Diatoms increased in abundance after 3 days in both treatments with nitrogen addition (N; N+Thi) and made up 30–40% of the community on Day 6. The diatoms consisted of mixed centrales, such as the genera *Chaetoceros*, *Coscinodiscus*, *Skeletonema* and *Melosira*, and pennales, represented by the genera *Nitzschia*, *Diatoma* and *Surirella*. Until the final sampling (Day 16), for small flagellates and Nostocophyceae, the abundance of filamentous cyanobacteria (mainly *Aphanizomenon flos-aquae*) increased to 30% in N and N+Thi and to 20% in C and Thi (Fig 3A). Phytoplankton biomass (mg C m^-3^) was dominated by the ciliate group Litostomatae at Days 0 and 3 in all treatments, after which this group decreased (Fig 3B). Litostomatae is classified as a phytoplankton since it consists of only the kleptoplastic species *Mesodinium rubrum*, which relies >95% on photosynthetic carbon acquisition [102]. On Day 6, Chlorophyceae, Diatomophyceae and Dinophyceae made up >50% of the biomass in all treatments and, together with small flagellates and Nostocophyceae, replaced Litostomatea on Day 16 (Fig 3B). Thiamin addition did not result in greater phytoplankton abundance at the different time points, while nutrient addition did (LMM, thiamin addition: χ^2^ = 1, p = 0.33; nitrogen addition: χ^2^ = 8.8, p<0.01; timepoint χ^2^ = 1.7, p = 0.19, no other interactive effects p>0.05). The same pattern was observed for biomass (LMM, thiamin addition: χ^2^ = 0.2, p = 0.63; nitrogen addition: χ^2^ = 15.6, p<0.01; timepoint χ^2^ = 21.4, p = <0.01, no other interactive effects p>0.05). The succession and within-treatment variation of the phytoplankton community separated over time rather than by treatment (Fig 5A). Days 0 and 3 separated from Days 6 and 16 along NMDS1, driven by changes in Litostomatea (p<0.05) abundance and biomass. Therefore, the analysis of the phytoplankton community using 16S chloroplast sequences, 18S rRNA sequences and microscopic counts suggests that nitrogen addition affected the phytoplankton community composition to some extent, whereas thiamin addition had no major effect.

### Zooplankton community composition

**Fig 4:**
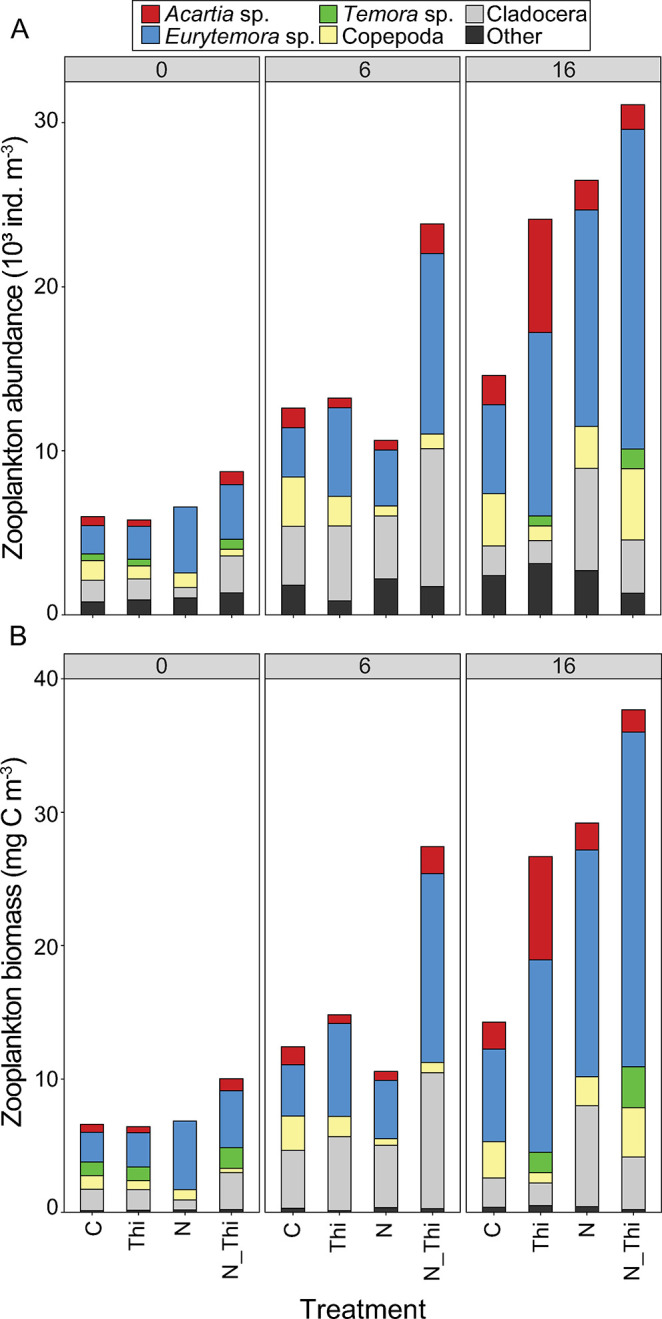
Total zooplankton abundance (10^3^ individuals m^-3^) (A) and biomass (mg C m^-3^) (B) for the dominant taxonomic groups in the Control (C), Thiamin (Thi), Nitrogen (N), and Thiamin + Nitrogen (N_Thi) treatments. There were overall higher abundances following N addition and a tendency toward higher abundances in response to thiamin addition (see statistics in the text). There were no significant differences among treatments in either abundance or biomass within each sampling occasion, i.e., on Days 3, 6 and 16 (Mann‒Whitney U test, multiple comparisons, p>0.05).

The zooplankton community composition was dominated by Copepoda (primarily *Eurytemora* sp.) in all the treatments and sampling occasions (Fig 4A). Some Cladocera and Balanus were also present. The molecular identification of individual zooplankton yielded consistent results with the microscopic identification of *Eurytemora affinis*, *Amphibalanus improvisus* and *Acartia bifolosa*. Individuals identified microscopically as Podon, Evadne, Temora and cyclopoids generally did not amplify or yielded low <90% nucleotide identity similarity in GenBank (S1 Table S2 in S1 File).

**Fig 5:**
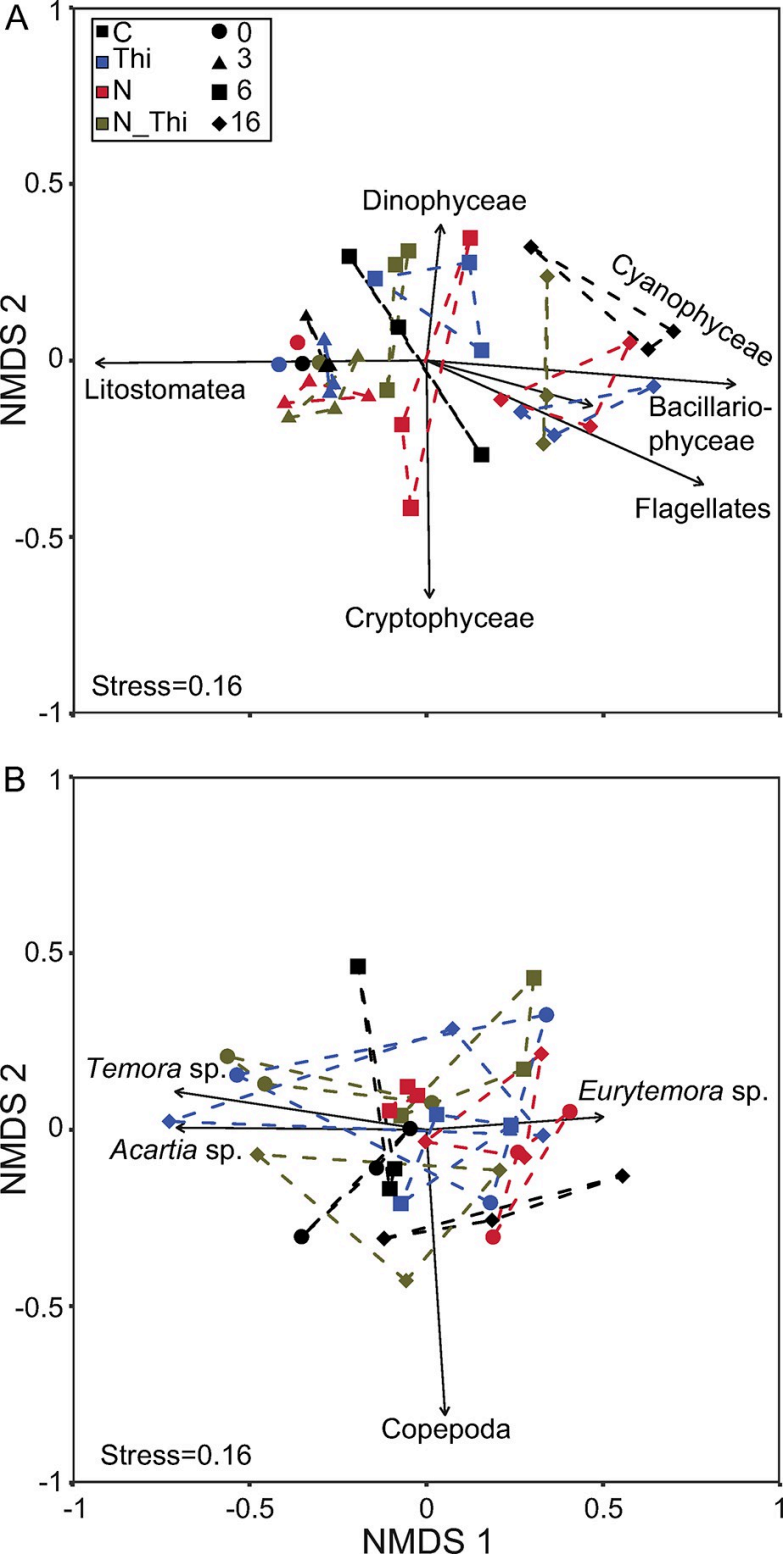
Nonmetric multidimensional scaling (NMDS) plot of the community composition of phytoplankton (A) and zooplankton (B). Each symbol represents the median community composition (Bray‒Curtis dissimilarity) for each replicate and time point (0, 3, 6, 16), while the color represents treatment; Control (C), Thiamin (Thi), Nitrogen (N), and Thiamin + Nitrogen (N_Thi). Dashed lines connect replicates within a time point and treatment. Arrows illustrate fitted vectors.

In the zooplankton community, there was generally a greater abundance in nitrogen addition treatments and a tendency toward greater abundance in thiamin addition treatments (LMM, thiamin addition: χ^2^ = 3.0, p = 0.09; nitrogen addition: χ^2^ = 5.2, p = 0.02; timepoint χ^2^ = 0.3, p = 0.56; no other interactive effects p>0.05). In addition, there were significant differences among the separate zooplankton groups and species (Fig 4A). Overall, the abundance of Copepoda was significantly greater in the treatments with nutrient addition (ANOVA (all F values below), F_(1,31)_ = 4.20, p<0.05), thiamin addition (F_(1,31)_ = 4.89, p<0.05) and among time points (F_(2,31)_ = 17.72, p<0.001,) but there were no interaction effects (LMM, χ^2^ = 0.31, p = 0.74). For Cladocera, the abundance was greater in the treatments with nutrient addition (F_(1,31)_ = 4.12, p<0.05) but not in the treatments with thiamin addition (F_(1,31)_ = 0.01, p = 0.92). Additionally, the Cladoceran abundance increased with time (F_(2,31)_ = 7.10, p<0.01), but there was no interaction effect between nutrient addition and time (χ^2^ = 2.01, p = 0.16). For separate copepod species, *Eurytemora* was present in significantly greater numbers in the treatments that received nutrient addition (Fig 4; F_(1,31)_ = 11.15, p<0.01) and thiamin addition (F_(1,31)_ = 7.95, p<0.01). The number of *Eurytemora* also increased significantly between the time points (F_(2,31)_ = 19.44, p<0.001). For *Acartia* and *Temora*, no significant effects of nutrient or thiamin addition were detected. The variation in the full zooplankton community did not differ over time or among treatments (Fig 5B).

### Thiamin in phytoplankton

**Fig 6:**
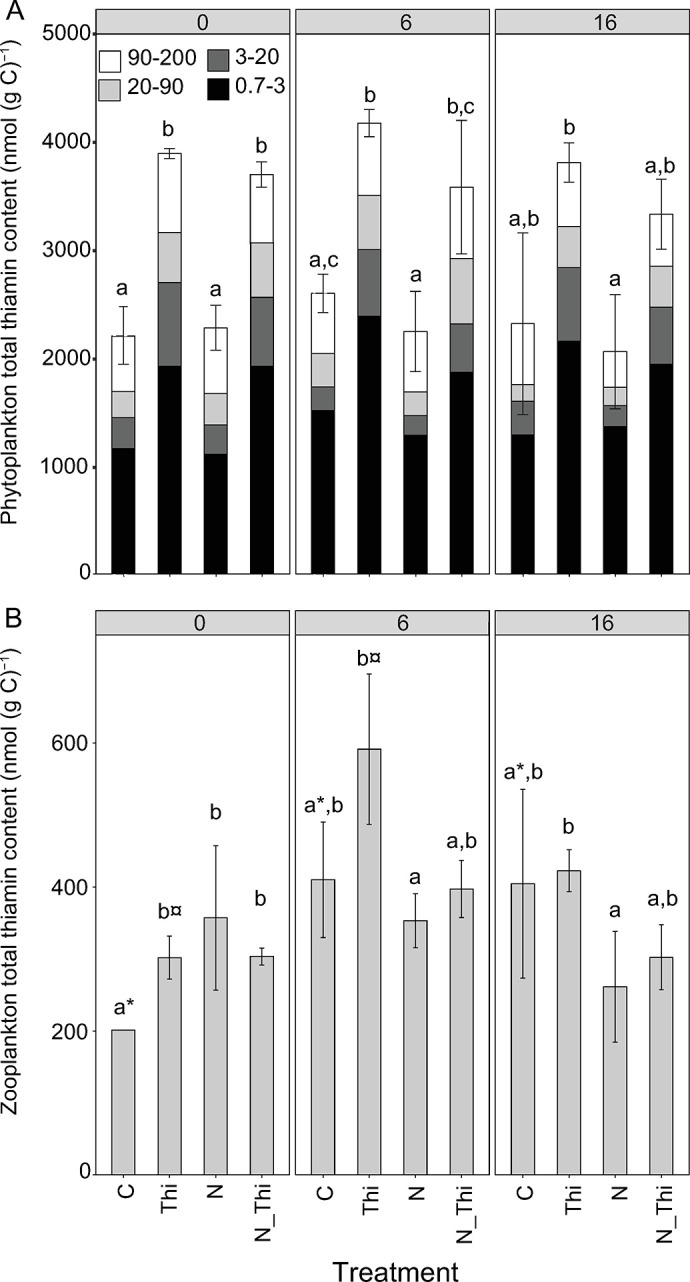
Total thiamin content (nmol (g C)^-1^) in phytoplankton (A) and zooplankton (B) in the Control (C), Thiamin (Thi), Nitrogen (N), and Thiamin + Nitrogen (N_Thi) treatments. Phytoplankton thiamin content is presented for separate size fractions (90–200 μm, 20–90 μm, 3.0–20 μm, and 0.7–3.0 μm). The error bars show the standard deviation of the total thiamin content. Different letters indicate significant differences among treatments within time points, while symbols indicate significant differences among time points within treatments.

Thiamin added at a concentration of 296.5 nM to Thi and N+Thi replicates was quickly (within hours) taken up by phytoplankton at the beginning of the experiment, and elevated thiamin levels in phytoplankton (i.e., particulate thiamin) were maintained in the Thi and N+Thi treatments throughout the entire experiment (Fig 6A). The concentrations of particulate thiamin in phytoplankton at the first time point, a few hours after the addition of dissolved thiamin, suggest that on the order of 69 ± 1.3% and 79.6 ± 4.5% of the dissolved thiamin was absorbed by the phytoplankton in the Thi and N+Thi treatments, respectively (based on biovolume estimates of phytoplankton). There was no significant interaction effect between sampling time and treatment for the phytoplankton total thiamin content when all size fractions were combined (LMM, χ^2^ = 0.43, p = 0.94). Therefore, the phytoplankton total thiamin content displayed the same general pattern among the treatments at the different time points. Overall, the thiamin content was significantly lower in the C treatments than in the Thi and N+Thi treatments but not in the N treatment (quantified on Days 0, 6 and 16; N: t = 1.07, p = 0.71; Thi: t = 6.92, p<0.001; N+Thi: t = 5.37, p<0.001). Additionally, the thiamin content was significantly lower in the N treatment group than in both the Thi and N+Thi treatment groups (Thi: t = 8.24, p<0.001; N+Thi: t = 6.6, p<0.001). Overall, the thiamin content was similar in the Thi and N+Thi treatments (t = 1.6, p = 0.39).

There were also differences in the total thiamin content at each separate sampling. On Day 0, the phytoplankton total thiamin content was greater in the Thi and N+Thi treatments than in both the C (Thi: t = 8.84, p<0.001; N+Thi: t = 8.04, p<0.001) and N (Thi: t = 9.33, p<0.001; N+Thi: t = 8.43, p<0.001) treatments (Fig 6A). By Day 6, the thiamin content was still greater in the Thi treatment group than in the C (t = 4.58, p<0.01) and N (t = 6.08, p<0.01) treatment groups, while the thiamin content in the N+Thi treatment group was only significantly greater than that in the N treatment group (t = 4.50, p<0.01; Fig 6A). On Day 16, the phytoplankton total thiamin content was significantly greater in Thi than in N (t = 3.20, p<0.05), while C and N+Thi had similar levels (Fig 6A).

The thiamin content in plankton of different sizes was quantified to explore the trophic relationships (Fig 6A). The smallest size class (0.7–3.0 μm) had by far the highest thiamin concentration compared with all other size classes. Overall, for all size classes, the thiamin levels were greater in the Thi and N+Thi treatments than in the C and N treatments, respectively, with few differences between the C treatment and the N treatment or between the Thi treatment and the N+Thi treatment (Fig 6A).

### Thiamin in zooplankton

For zooplankton, there was no consistent treatment effect of either thiamin or nitrogen addition on the total thiamin content. Thiamin content displayed an interaction effect between sampling time and treatment (LMM, χ^2^ = 19.12, p<0.01). Therefore, the general pattern of the thiamin content in the different treatments differed among the samples. In the control treatment, the thiamin content was lowest on Day 0 (t test; applies to all “t” below; Day 6: t = 5.28, p<0.001; Day 16: t = 5.01, p<0.001) but was similar between Day 6 and Day 16 (t = 0.30, p = 1; Fig 6B). The thiamin content was similar at all sampling times for the N and N+Thi treatments. In the Thi treatment group, the thiamin content was significantly greater on Day 6 than on Day 0 (t = 4.90, p<0.001,) but the difference was similar in the other comparisons (Fig 6B). When investigating separate samplings, the pattern varied somewhat. On Day 0, the total zooplankton thiamin content was significantly lower in the C treatment group than in the remaining treatment groups (N: t = 4.27, p<0.01; Thi: t = 3.34, p<0.05; N+Thi: t = 3.38, p<0.05), which had similar thiamin contents (Fig 6B). On Day 6 and Day 16, the total zooplankton thiamin content was significantly greater in the Thi treatment group than in the N treatment group (Day 6: t = 3.75, p<0.01; Day 16: t = 3.77, p<0.01) (Fig 6B). Thiamin concentrations in zooplankton were on the order of 10–20% of the concentrations in phytoplankton. The chain-forming diatom Melosira was present in the >200 μm fraction but only at negligible concentrations compared to the zooplankton biomass.

The similar concentrations of thiamin per biomass but different abundances of zooplankton among the treatments (Fig 4) suggest that the total amount of thiamin trapped in the zooplankton biomass differed among the treatments. This is illustrated by the amount of thiamin extracted from zooplankton per L of water, which was 21.1 ± 6.8, 32.8 ± 9.6, 28.3 ± 2.0, and 42 ± 6.3 pM in C, Thi, N and N_Thi, respectively (Day 16). This corresponds to 56, 34 and 100% greater thiamin amount trapped in the total zooplankton biomass in the N, Thi and N+Thi treatments, respectively, compared to the C treatment. Therefore, the total amount of thiamin trapped in zooplankton differed among the treatments (ANOVA; F_3,8_ = 5.1, p = 0.03). Multiple comparisons revealed differences between C and N+Thi (Tukey test, p = 0.02,) but no other statistically significant differences were detected (p>0.05).

### Thiamin concentrations at different trophic levels in comparison to mesocosm concentrations (data collected from the literature)

**Fig 7:**
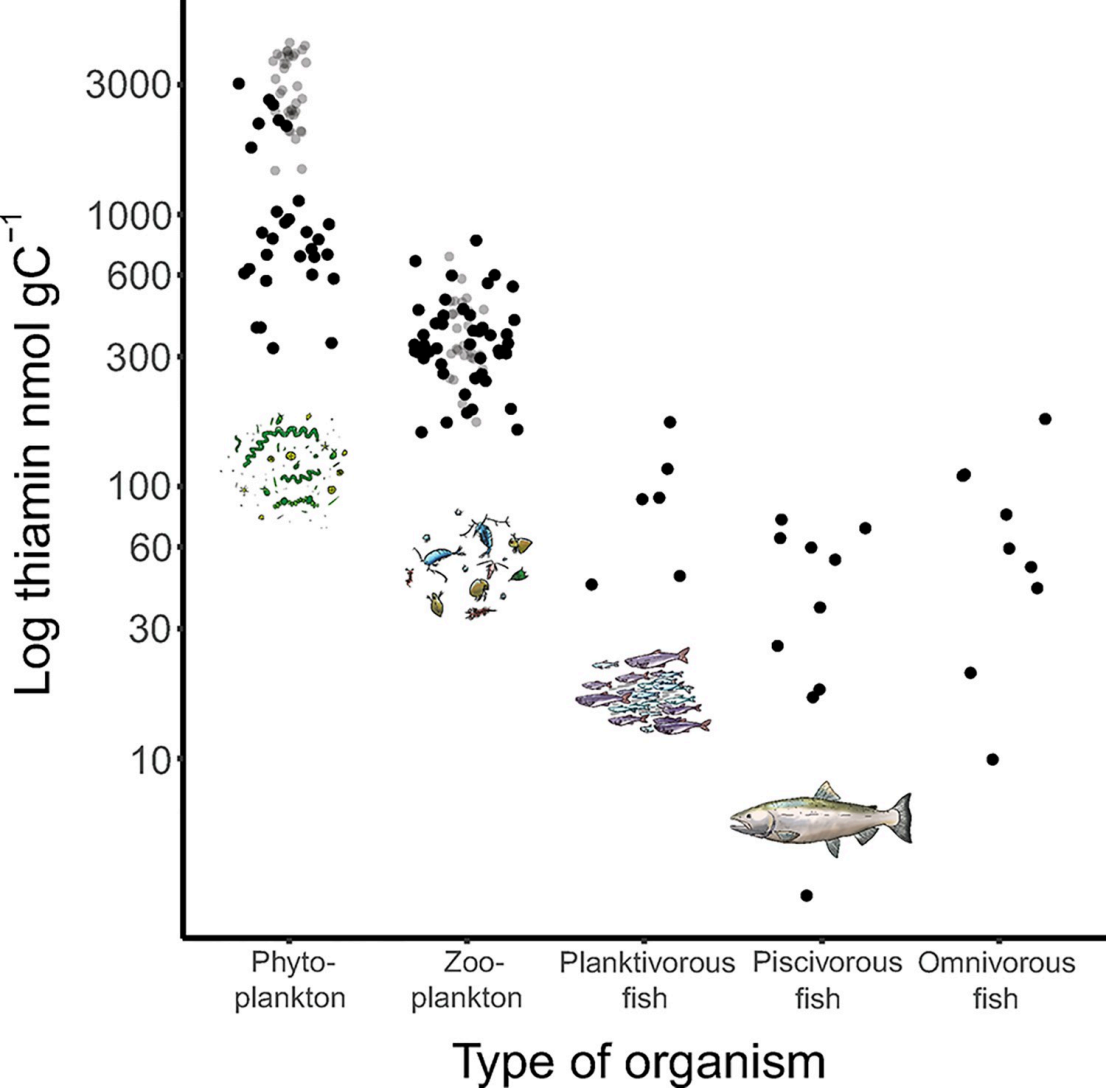
Total thiamin content (log; nmol gC^-1^) in phytoplankton, zooplankton, planktivorous fish, piscivorous fish and omnivorous fish (drawings modified from [52]). Data collected in a literature search (black dots) on the concentrations of thiamin in different organisms (phytoplankton, zooplankton, planktivorous fish, piscivorous fish, and omnivorous fish). The grey dots represent mesocosm concentrations of phytoplankton and zooplankton.

Literature data on thiamin concentration per unit of carbon were used to characterize the trophic transfer of thiamin from phytoplankton via zooplankton and planktivorous fish to top consumers, such as piscivorous fish. This analysis revealed that the average and median thiamin concentrations were highest in phytoplankton (seston), followed by zooplankton, omnivorous fish, and planktivorous fish, and the lowest concentrations were detected in piscivorous fish (Table 1, Fig 7). There was a gradual linear decline in log concentrations from lower to higher trophic levels when comparing phytoplankton, zooplankton, planktivorous fish and piscivorous fish (linear regression, F_3,45_ = 68.7, p<0.05, linear equation y = -1.05x + 6.8). Approximately 6% of the thiamin in phytoplankton occurs in piscivorous fish (Table 1, Fig 7). Omnivorous fish were not included in the linear regression since they feed from several trophic levels. Thiamin concentrations varied considerably in phytoplankton, ranging from 323–3,035 nmol gC^-1^. There was less variation within zooplankton, ranging from 159–804 nmol gC^-1^; planktivorous fish, from 44 to 173 nmol gC^-1^; piscivorous fish, from 3–76; and finally omnivorous fish, ranging from 10–177 nmol gC^-1^ (Table 1, Fig 7). Estimates of thiamin concentrations in zooplankton in the mesocosm study (gray dots, Fig 7) were in the same range as the literature data, whereas thiamin concentrations in phytoplankton were at the higher end or higher than those observed in field data (gray dots, Fig 7, compare with Fig 6 for the different N and thiamin amendment treatments).

**Table 1:** Summary of the median, average, maximum and minimum concentrations of total thiamin in phytoplankton (or seston), zooplankton, planktivorous fish, piscivorous fish and omnivorous fish.

Type of organism	Median total thiamin (nmol gC^-1^)	Average total thiamin (nmol gC^-1^)	Max total thiamin (nmol gC^-1^)	Min total thiamin (nmol gC^-1^)	Loss in food web (%)	Loss each trophic step (%)
Phytoplankton	813	1093	3035	323	Producers	Producers
Zooplankton	333	359	804	159	41	59
Planktivorous fish	90	93	173	44	11	73
Piscivorous fish	45	42	76	3	6	50
Omnivorous fish	59	73	177	10	7	*

The loss of thiamin along the food web (i.e., % remaining in that trophic level compared to that of phytoplankton), as well as the loss in each trophic step (i.e., % lost from the previous trophic level), is calculated assuming 100% in phytoplankton. Loss in each trophic step is not calculated for omnivorous fish since they feed from several trophic levels (indicated by *).

## Discussion

This study demonstrated that dissolved thiamin was readily absorbed by primary producers, but interestingly, there were no major shifts in the phytoplankton community composition in response to thiamin treatment. Nitrogen addition led to major shifts in abundance and some changes in community composition but without any major additional effects of combined nitrogen and thiamin addition. High concentrations of thiamin in phytoplankton did not directly lead to high concentrations in zooplankton. However, thiamin addition increased the abundance of some zooplankton species that are important prey for small fish in this system, suggesting overall increased food web transfer of this micronutrient (especially in the combined nitrogen and thiamin addition treatment). Therefore, this increase in the absolute amount of thiamin in the total zooplankton biomass was not driven by increasing concentrations but rather by an increase in the abundance of certain zooplankton upon thiamin and nitrogen amendment. Our literature summary of thiamin concentrations at different trophic levels revealed levels similar to those in the mesocosm study. There was a trophic dilution of approximately 6% of the thiamin concentration in the phytoplankton present in the top-predatory fish.

### Thiamin uptake in plankton

Total thiamin concentrations in phytoplankton in the mesocosm experiment varied by approximately a factor of two among treatments. This is in line with previous amendment experiments that have shown that phytoplankton take up dissolved thiamin or its metabolically relevant biosynthetic precursors and degradation products from water [32–35, 40].

Interestingly, thiamin addition did not have any major effect on the overall biomass production of heterotrophic picoplankton or phytoplankton. There was a slight increase in autotrophic picoplankton during some parts of the study in response to thiamin addition. However, in line with other studies [11, 103], nitrogen addition led to much greater effects, with greater microbial biomass production all the way from bacteria to zooplankton, but without any single or additive strong effect of thiamin supplementation. Our results contrast with those of other studies suggesting that B vitamins can limit overall phytoplankton growth in natural communities [13, 14, 104, 105]. Phytoplankton species known to be auxotrophs can exhibit increased growth upon thiamin amendment [41], but we did not observe any shifts in community composition in response to thiamin addition. The mechanisms maintaining stable community composition are not known, but one can speculate that a few dominant species were not in need of additional thiamin, whereas a few other less dominant species were responsible for the uptake without increasing relative abundance significantly. The affinity of the studied food web for dissolved thiamin was high, with significantly higher particulate thiamin (i.e, in plankton) concentrations within a few hours after the addition of dissolved thiamin. Comparing the estimated concentration in water to that in phytoplankton (by biovolume) suggested that 70–80% of the added dissolved thiamin was absorbed by phytoplankton to become particulate thiamin. Some of the dissolved thiamin may also have been absorbed by the biofilm that usually forms on the walls of mesocosms (not estimated).

Several mechanisms, including exposure to solar radiation, high temperature and high pH, have been shown to reduce the availability of dissolved thiamin [56, 57, 106]. Therefore, there was likely natural degradation and removal of dissolved thiamin with time, but the increase in particulate thiamin in phytoplankton was largely maintained throughout the entire mesocosm experiment, suggesting strong initial uptake and then selection for the reuse of excreted thiamin. The pH was greater in the N- amended treatments, which could indicate greater pH-induced loss of dissolved thiamin in those treatments, but we did not observe any evidence of reduced uptake in response to pH, with similar levels of particulate thiamin in the Thi treatment group compared to the N+Thi treatment group.

### Plankton community composition and thiamin

Evidence is now emerging that both thiamin producers and thiamin auxotrophs (full or partial) are widespread among bacteria and phytoplankton [37, 38, 40]. Amendment of thiamin increases concentrations in both auxotrophs and thiamin-producing phytoplankton species [41], which may indicate that there is a high affinity for this molecule regardless of the ability to produce thiamin. It is also evident in the literature that thiamin concentrations in phytoplankton are species-specific, and studies of laboratory isolates have shown that thiamin concentrations are greater in filamentous cyanobacteria than in a range of green algae, diatoms, dinoflagellates and cryptophytes [41, 42]. In the present study, the community compositions of phytoplankton and bacteria were affected by nitrogen addition but were interestingly similar in the thiamin and nonthiamin treatments. This result suggests that an increase in dissolved thiamin did not lead to major changes in competition patterns among species in the present primary producer community. Both bacteria and phytoplankton have been shown to be able to regulate thiamin production with the help of so-called riboswitches [36, 39]. This observation suggests that producers can switch to the uptake of dissolved thiamin when available and utilize the full thiamin molecule or different combinations of its precursors and analogues [32–35].

### Availability of dissolved thiamin in natural systems

The overall availability of dissolved thiamin, its precursors and analogues in natural systems is not well known, but estimates worldwide suggest that it ranges from nondetectable to pM concentrations [13, 43, 44] and there are no published measurements of dissolved thiamin in the Baltic Sea to compare with. Therefore, the amount of dissolved thiamin added in this experiment was likely greater than that usually found in marine waters, providing an ample source of thiamin to organisms. Phytoplankton indeed took up thiamin, and particulate thiamin concentrations increased significantly.

### Thiamin in zooplankton

The concentrations of thiamin in zooplankton were less variable than those in phytoplankton. This is in line with previous estimates from laboratory and field conditions [41, 45, 47]. For example, Fridolfsson et al. (2019) showed that per carbon thiamin concentrations in zooplankton are relatively stable throughout different seasons at concentrations approximately 50% of those of phytoplankton. This observation suggests that differences in thiamin concentrations in phytoplankton are not manifested in a greater trophic transfer to zooplankton. The mechanism for this could be that zooplankton, especially copepods, are size selective in their feeding [107] and could avoid some large thiamin-rich phytoplankton, such as filamentous cyanobacteria, and instead rely on other prey, such as diatoms and ciliates [47]. Although they are thiamin rich, large filamentous cyanobacteria have been shown to be detrimental to copepod thiamin concentrations and egg production (Fridolfsson et al. 2018). An alternative explanation could be that zooplankton are independent of thiamin concentrations in their diet and are entirely dependent on their gut microbiome for thiamin provision. This topic has not been studied extensively in zooplankton, but recent studies in *Drosophila* suggest that this taxon is dependent on thiamin provided by the gut microbiota [29].

While this and previous studies have suggested that zooplankton are relatively stable in terms of their thiamin concentration, we demonstrate here that thiamin addition led to a greater abundance of certain copepod taxa. The mechanisms for this pattern are not known. Other studies have shown that increased food quality can lead to increased offspring production among copepods. Food quality can be reflected in biochemical or stoichiometric composition as well as the level of digestibility of the prey [108]. In particular, highly unsaturated fatty acids have been shown to be crucial for the biochemical composition of zooplankton [108, 109], whereas vitamins and their effects on copepod prey have been less studied in zooplankton. Copepods increased in general, and in particular, the most common species, *Eurytemora affinis*, increased in abundance following thiamin treatments. This result was also supported by a greater relative abundance of Arthropoda amplicons at the end of the mesocosm study. This copepod species is a key prey item in the Baltic food web [110, 111], suggesting that changes in the availability of dissolved thiamin could lead to a greater absolute amount of thiamin available for planktivorous fish. The absolute amount of thiamin trapped in zooplankton biomass indeed differed among treatments, with an especially greater absolute amount in the combined thiamin and nitrogen addition treatment than in the control. This effect was driven by a higher abundance of zooplankton in some treatments and not by a higher per carbon concentration of thiamin. Other common zooplankton taxa, such as cladocerans, were not stimulated by thiamin addition, suggesting other mechanisms for their biomass production.

### Food web transfer

By comparing the results from the mesocosm study with literature data on thiamin at different trophic levels, we demonstrated that thiamin is diluted as it is transferred in the food web from lower to higher trophic levels. The concentrations of thiamin in the mesocosm study were generally similar to those reported in the literature among zooplankton. The concentrations in phytoplankton in the mesocosm study were in the highest end in the literature but in the same order as the summer concentrations of phytoplankton in the Baltic Sea (often dominated by thiamin-rich cyanobacteria) [45].

The trophic transfer of macronutrients from producers to top consumers is well known [6, 7, 11, 103], but less is known about micronutrients, especially organic micronutrients such as vitamins, and their transfer from lower to higher trophic levels, as studied here [9, 10]. Thiamin has mainly been studied among top consumers, such as fish and birds, and among producers, such as bacteria and phytoplankton, with less knowledge on intermediate consumers, such as zooplankton [41, 47, 112]. There is also little information regarding the ecological processes leading to the transfer of vitamins throughout the entire food chain from lower to higher trophic levels [113]. The main route of vitamin transfer is through the diet or by provision from the gut microbiota. In humans, the microbiota is capable of producing up to 30% of the daily need for B_2_, B_3_ and B_12_ vitamins [114], with some possibility of providing thiamin [18, 115, 116]. In mammals, dietary vitamins are absorbed in the small intestine, and the uptake of microbially produced vitamins occurs in the large intestine [117]. Therefore, the *de novo* synthesis of thiamin is likely a significant route in herbivores, such as ruminants or herbivores in other groups that have a large colon and a time-consuming digestion [27, 28, 30]. However, carnivory in fish leads to shortened digestive tracts [31], potentially reducing the space in which microbiota can produce thiamin *de novo* for later provision to the host [27, 28]. The role of the gut microbiota in the thiamin supply of aquatic herbivores such as zooplankton is unknown. The aquatic food web consists of small herbivores (zooplankton) and fish that rely on carnivory which suggest that diet is a main route for thiamin supply and transfer from lower to higher trophic levels. However, the main mechanisms leading to dilution are not known but could include sloppy feeding, suboptimal uptake due to thiaminase or other issues in the gut, more efficient metabolism at higher trophic levels leading to a reduced need for thiamin, or other unknown factors.

This flow of thiamin in the food web has previously been modeled from microbes, via zooplankton, up to planktivorous fish, with a size-structured food web approach and a bioavailability (proportion absorbed from food) per trophic level assumed to be in the range of 10–20% [118]. Our analysis of literature data showed that bioavailability at each trophic step was 33% for zooplankton when feeding on phytoplankton and 20% for planktivorous fish feeding on zooplankton, which is within and above the upper range of bioavailability assumed in the Ejsmond et al. (2019) food web model. This food web modeling concluded that with a thiamin bioavailability of 20% per trophic level, approximately 1.6‰ to 4% of the thiamin produced by picoalgae and microalgae, respectively, reaches planktivorous fish [118]. Overall, our analysis of literature data showed that the concentration of thiamin in piscivorous fish was equal to 6% of the available pool in phytoplankton. The bioavailability of thiamin for piscivorous fish feeding on planktivorous fish was particularly high, reaching 75% (Table 1). This result suggests that thiamin bioavailability can be greater than previously assumed [118], yet only a small percentage of the thiamin pool available in primary producers actually reaches top consumers. These estimates can also be community composition-specific since not all prey are readily consumed at the next trophic level. For example, filamentous cyanobacteria are rich in thiamin, but a relatively small proportion of this thiamin is transferred to zooplankton [41]. Zooplankton feeding onother prey, such as R. *salina*, with lower thiamin concentrations are still able to sustain approximately the same thiamin concentration [41].

### Development of thiamine deficiency

As thiamin is transferred in the food web to top consumers, there are several examples of thiamin deficiency. For example, common eider populations in the Baltic Sea [20, 24, 119] and salmonids in several systems worldwide have at times displayed neurological symptoms and negative effects on reproduction, which could be mitigated by thiamin supplementation [9, 21, 22, 25]. Several mechanisms leading to thiamin deficiency in wild animal populations have been proposed, including changes in trophic transfer, the occurrence of thiamin-degrading enzymes, or a diet rich in polyunsaturated fatty acids [22, 46, 50–52]. This study demonstrates that there is a strong trophic dilution of thiamin from producers to top consumers, suggesting that changes in the lower part of the food web could affect the thiamin status at higher trophic levels. This is exemplified by the increased abundance of some copepod species upon thiamin addition in the mesocosm study. Our results are also consistent with conclusions from food web modeling [118], with nutrient levels mainly affecting thiamin transport in the food web due to shifts in functional group biomass rather than changes in thiamin concentration in organism cells/bodies.

Therefore, we propose that producers can have a wide range of thiamin concentrations, whereas all consumer levels (zooplankton, planktivorous and piscivorous fish) have a much lower variation of thiamin within the trophic level. Consumers have been shown to maintain stable thiamin homeostasis [45, 112]. Similarly, macronutrient homeostasis is more common among consumers than among primary producers [5, 120]. Therefore, some of the trophic dilution observed in this study could be driven by more efficient metabolism in multicellular organisms than in primary producers. This could enable consumers to operate on a lower thiamin-to-carbon quota than primary producers. However, thiamin requirements among different fish species vary [121–127], suggesting that there is also some variation in thiamin to carbon ratios among species within the same trophic level.

The major pathways leading towards thiamin deficiency due to changes in the food web should therefore be via elongated food webs where more trophic levels are included, for example, if thiamin is produced and fueled via the microbial loop with more trophic levels compared to the traditional phytoplankton, zooplankton, small fish and top predator food chain [46]. Other pathways leading to deficiency include long periods of starvation, the use of a monotrophic diet that does not provide enough thiamin, a reduced supply via the gut microbiota, reduced uptake due to thiaminase, or a skewed ratio of thiamin to other nutrients, e.g, nitrogen or carbon, in the diet. A tendency toward the latter mechanism was observed in the mesocosm study (albeit not significant), with slightly lower concentrations of thiamin among zooplankton in the nitrogen treatments on the final sampling occasion. Similarlyto the shifting thiamin to nutrient ratio effects studied here other studies suggest that a diet rich in polyunsaturated fatty acids can cause thiamin deficiency in salmon [50, 51]. However, to understand the underlying mechanisms, more information is needed regarding the variation in thiamin concentrations among species and individuals. This is especially important in planktivorous fish, as these are the main prey items for top consumers displaying thiamin deficiency, and many previous studies have been performed with pooled samples of several individuals, reducing the possibility of assessing the variability in thiamin availability per prey item. In addition, other pathways leading to deficiency that need further study include reduced uptake in the intestines due to the occurrence of thiaminase [22] or decreased availability of vitamins from the gut microbiota, which could be relevant as we revealed a relatively small reduction in the thiamin pool in large fish compared to that in small fish species.

### Conclusions

Here, we demonstrate that thiamin concentrations are highest in the smallest organisms and that there is a gradual trophic dilution as the molecule is transferred up the food web. Thiamin supplementation led to rapid uptake in the phytoplankton without any major shifts in community composition, regardless of nitrogen addition. Higher thiamin concentrations in phytoplankton were not reflected in zooplankton. However, some zooplankton were stimulated by thiamin addition, leading to higher abundances. Overall, these findings suggest that changes in the thiamin pool in the lower parts of the food web can affect thiamin availability at higher trophic levels, but the underlying mechanisms need further study.

## Supporting information

S1 File Word file with additional methods and results as well as supplemenatry figures and tables. (DOCX)

S2 File Excel file with the raw data underlying the manuscript. (XLSX)
