# Supplementary material for: Thiamin (vitamin B1, thiamine) transfer in the aquatic food web from lower to higher trophic levels
Source: PLoS One. 2024 Dec 2;19(12):e0308844. doi: 10.1371/journal.pone.0308844 (PMC11611157; doi:10.1371/journal.pone.0308844)
Supplement: S1 File — (DOCX) [file pone.0308844.s001.docx]

**Supplementary material**

**Thiamin (vitamin B1, thiamine) transfer in the aquatic food web from lower to higher trophic levels**

Samuel Hylander^1,*^, Hanna Farnelid^1^, Emil Fridolfsson^1^, Marc M. Hauber^1^, Vittoria Todisco^1^, Maciej J. Ejsmond^1,2^ and Elin Lindehoff^1^

^1^Department of Biology and Environmental Science, Centre for Ecology and Evolution in Microbial Model Systems (EEMiS), Linnaeus University, Kalmar, Sweden.

^2^Institute of Environmental Science, Faculty of Biology, Jagiellonian University, Cracow, Poland.

^*^Corresponding author: Samuel.Hylander@lnu.se

# **Content and structure of the supplementary material S1-2**

**S1**

S1- Additional methods with detailed information regarding DNA extractions and thiamin analysis

S1 - Additional results on nutrients and thiamin concentrations in different size classes of phytoplankton. This includes supplementary figures S1-S2.

S1 Figs. S1-5: Supplementary results and figures (S1-5) on community composition of 16S rRNA chloroplasts,16S rRNA and 18S rRNA and experimental setup.

S1 Table S1 on number of high-quality reads (18S and 16S rRNA gene amplicons) and proportion of chloroplasts in 16S rRNA gene libraries per sample.

S1 Table S2 on number of identification of zooplankton using microscopy and two sets of primers.

**S2**

Supplementary file 2 (S2) - A separate excel file called S2 Table S3. This file contains all data associated with the manuscript (except the genetic data which is available at the Sequence Read Archive (SRA) with BioProject ID PRJNA1051975). The different sheets in this file include:

Abiotic factors: Temperature, salinity, pH, chlorophyll *a* and nutrients

Thiamin: Thiamin concentrations in phytoplankton and zooplankton

Particulate C and N: Particulate concentrations of carbon and nitrogen in phytoplankton and zooplankton.

Zooplankton abundance: Zooplankton counts in stereomicroscope

Heterotrophic/autotrophic cells: Results from cell counts using flow cytometry

Phytoplankton: Phytoplankton cell counts using microscope.

# **S1 - Additional methods**

## *DNA extractions -* Briefly, Supor filters were thawed and cut into small pieces using sterile scissors and placed into bead tubes with 978 µl Sodium Phosphate buffer and 122 µl MT buffer. Cells were homogenized three times using the FastPrep® instrument (Fastprep-24 5G, MP Biomedicals) for 40 s at a speed setting of 6.0 m/s followed by incubation with proteinase K (1% final concentration) at 55°C for 1 h. To remove cell debris, tubes were centrifuged at 14,000 x g for 15 min and the supernatant was transferred to a new tube and mixed with 250 µl Protein Precipitation Solution. The solution was centrifuged at 14,000 x g for 5 min and the supernatant was transferred to a 15 ml falcon tube with 1 ml of Binding Matrix Suspension. Tubes were shaken by hand for two minutes and the mixture was added to a SPIN™ Filter and centrifuged at 14,000 x g for 1 min. The matrix was washed using 500 µl SEWS-M washing solution and centrifuged at 14,000 x g for 1 min and the SPIN filters were dried in a subsequent centrifugation for 2,5 min. DNA samples were eluted using DNase/Pyrogen-Free Water and the quality and yields of the extractions were measured using Nanodrop 2000 (Thermo Fisher Scientific) and Qubit (Thermo Fisher Scientific) and stored at -20°C.

# **S1 - Additional results**

*Additional nutrient results -* Dissolved silica (SiO_3_) concentrations were initially in the range of 2.9-2.7 µM and decreased during the experiments in all treatments. The decrease, until day16, was larger in treatments with N additions, N and N+Thi (0.4-0.5 µM), compared to C and Thi 1.9-2 µM with a significant N addition by time interaction but not thiamine effect (Thiamine addition: χ^2^=1.0, p=0.3; Nitrogen addition: χ^2^=98.3, p<0.01; Time χ^2^=520.9, p<0.01, N:Time: χ^2^=137.6, p<0.01, no other interactive effects p>0.05). Ammonium NH_4_^+^ (0.2-0.4 µM), total P (0.6-0.9 µM) and total N (21.7-28.4 µM) (Fig. S2) was with in respective range during the experiment with slightly higher concentrations in response to N addition and slightly declining concentrations over time but no effect of thiamine addition (Thiamine addition: χ^2^=2.0, p=0.2; Nitrogen addition: χ^2^=6.6, p=0.01; Time χ^2^=4.8, p=0.03, no interactive effects p>0.05). Molar ratio of total nitrogen to total phosphorus indicate that the biomass had a surplus of nitrogen throughout the experiment (Fig. S2;. If only inorganic nutrient species are included, DIN (NO_2_^-^+NO_3_^-^+ NH_4_^+^) to DIP (PO_4_^3-^) the biomass is indicated to be in N limitation with ratios below 16 (Redfield 1958) (Fig. S2). Addition of N increased the DIN:DIP ration to 11.8±1.1 and 12.7±1 in N and N+Thi treatment respectively, compared to 1.2 to 1.4 in C and Thi treatments While the DIN:DIP ratio remained between 1.2 to 2.4 in C and Thi treatments, the DIN:DIP in N and N+Thi treatments decreased from the initial values to 3.4 to 3.6 at the end of the experiment (Fig. S2).

Comparisons of thiamin concentrations in phytoplankton among sampled size classes *-*

At day0 in the smallest size fraction (0.7-3 µm), total thiamin content was higher in Thi and N+Thi compared to C (Thi-C: t=8.48, p<0.001; N+Thi-C: t=8.46, p<0.001) and N (Thi-N: t=9.34, p<0.001; N+Thi-N: t=9.31, p<0.001).Treatments with thiamin addition had similar levels (t=0.03, p=1) as was the case for treatments with no thiamin addition (t=0.86, p=0.82). In the size fraction 3-20 µm, total thiamin content was also at similar levels in Thi and N+Thi (t=1.46, p=0.51) but higher compared to C (Thi-C: t=6.35, p<0.01; N+Thi-C: t=5.04, p<0.01) and N (Thi-N: t=7.39, p<0.001; N+Thi-N: t=5.93, p<0.01), which had similar thiamin content (C-N: t=0.26, p=1). The pattern was the same in the size fraction 20-90 µm, with similar thiamin content in Thi and N+Thi (t=0.77, p=0.86) as well as C and N (t=1.60, p=0.43). Thiamin content was higher in Thi and N+Thi compared to C (Thi-C: t=5.59, p<0.01; N+Thi-C: t=6.36, p<0.001) and N (Thi-N: t=3.98, p<0.05; N+Thi-N: t=4.76, p<0.01). In the largest size fraction (90-200µm) at day0, total thiamin content only differed significantly between two treatments, C and Thi (t=3.51, p<0.05) whereas the levels were similar among the remaining treatments (C-N: t=1.73, p=0.37; C-N+Thi: t=2.07, p=0.24; Thi-N: t=1.78, p=0.35; Thi-N+Thi: t=1.45, p=0.51; N-N+Thi: t=0.34, p=0.99).

The patterns were similar on day6, but in the smallest size fraction (0.7-3 µm) total thiamin content was only significantly different between Thi and N treatment (t=3.30, p<0.05), even if thiamin tended to be higher in the thiamin treatments compared to the treatments without thiamin addition (. For the size fraction 3-20 µm thiamin content was similar in Thi and N+Thi (t=1.95, p=0.28) as well as C and N (t=1.13, p=0.68). Thiamin content was higher in Thi and N+Thi compared to C (Thi-C: t=5.81, p<0.001; N+Thi-C: t=3.87, p<0.05) and N (Thi-N: t=6.95, p<0.001; N+Thi-N: t=5.00, p<0.01). In the next size fraction (20-90 µm), total thiamin content was similar in Thi and N+Thi (t=0.72, p=0.89) as well as C and N (t=1.17, p=0.66). Levels were also similar among Thi, N+Thi and C (C-Thi: t=2.35, p=0.16; C-N+Thi: t=3.08, p=0.06). However, total thiamin content was significantly lower in N compared to Thi (t=3.52, p<0.05) and N+Thi (t=4.24, p<0.05). In the largest size fraction (90-200µm) at day6, total thiamin content was significantly higher in Thi compared to C, (t=3.21, p<0.05) which was the only significant differences present as the levels were similar among the remaining treatments (C-N: t=0.01, p=1; C-N+Thi: t=2.93, p=0.07; Thi-N: t=3.20, p=0.051; Thi-N+Thi: t=0.28, p=0.99; N-N+Thi: t=2.92, p=0.07).

At day16 for the size fraction 0.7-3 µm, no significant differences in total thiamin content were present (ANOVA, F_(3,8)_=1.84, p=0.22). However, in the size fraction 3-20 µm thiamin content was higher in Thi and N+Thi compared to C (Thi-C: t=5.55, p<0.01; N+Thi-C: t=3.80, p<0.05) and N (Thi-N: t=8.48, p<0.001; N+Thi-N: t=6.73, p<0.001). Levels were similar in Thi and N+Thi (t=1.76, p=0.36) as well as C and N (t=2.93, p=0.07). This pattern was also true for the size fraction 20-90 µm with thiamin content being higher in Thi and N+Thi compared to C (Thi-C: t=4.25, p<0.05; N+Thi-C: t=4.15, p<0.05) and N (Thi-N: t=3.94, p<0.05; N+Thi-N: t=3.84, p<0.05). Also, levels were similar in Thi and N+Thi (t=0.11, p=1) as well as C and N (t=0.31, p=0.99). For the size fraction 90-200 µm, total thiamin content was significantly lower in N compared to C (t=6.93, p<0.001), Thi (t=7.62, p<0.001) and N+Thi (t=4.89, p<0.001), whilst no other significant differences were present (C-Thi: t=0.69, p=0.90; C-N+Thi: t=2.04, p=0.25; Thi-N+Thi: t=2.73, p=0.10).


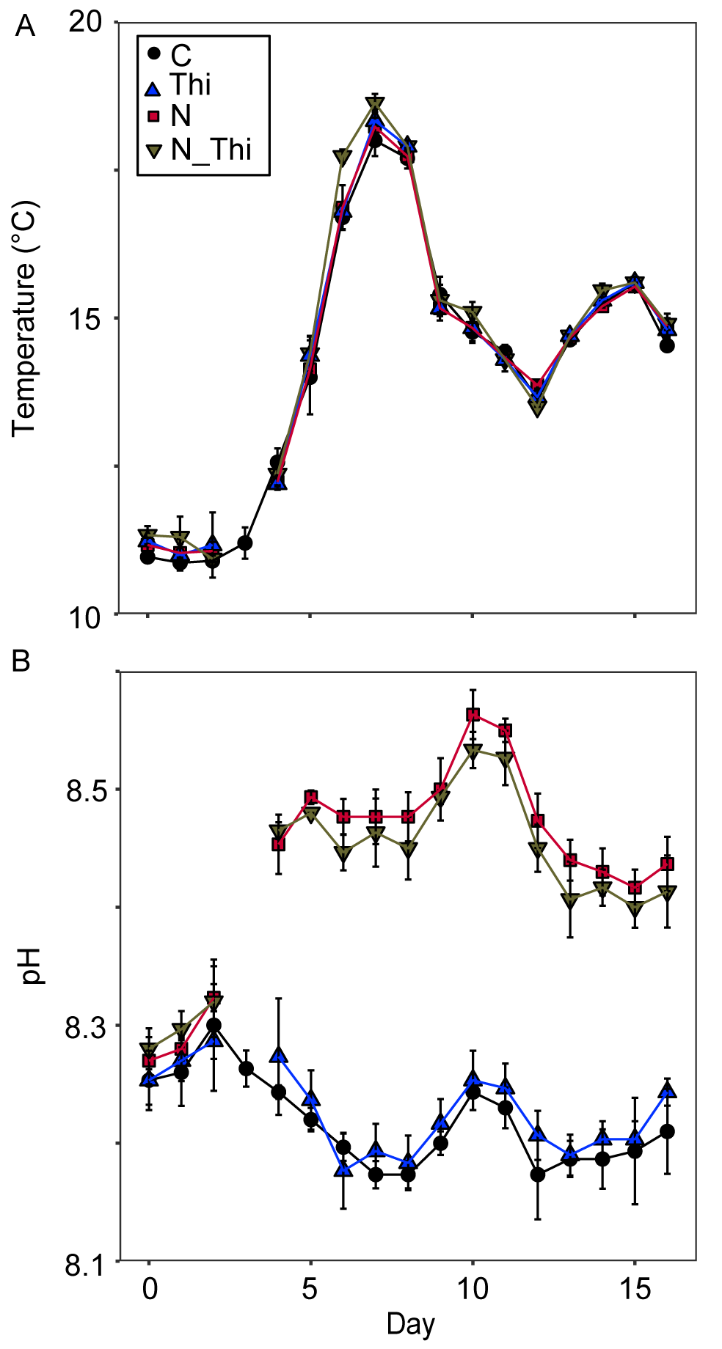


**S1 Figure S1.** Temperature (A) and pH (B) in the treatments; Control (C), Thiamin (Thi), Nitrogen (N), and Thiamin + Nitrogen (N_Thi) for each separate time point. Error bars show standard deviations for triplicates. The pH-meter was malfunctioning during sampling occasion number three so that data is not available for treatments Thi, N and N_Thi. For significant differences among treatments see the text.

**
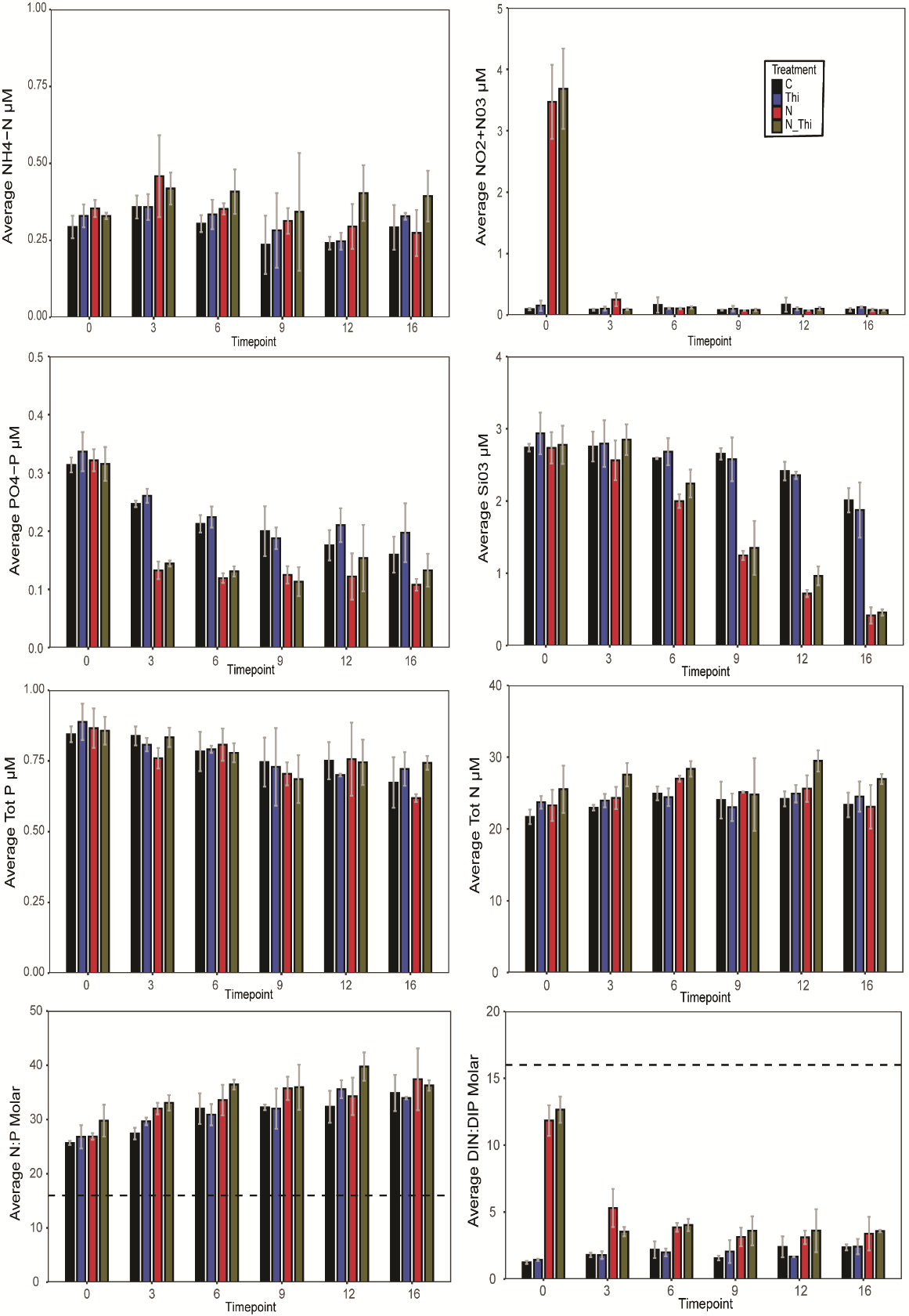
**

**S1 Figure S2.** Average NH4-N, NO2-NO3, PO4-P, SiO3, TotP, TotN, N:P ratio and DIN:DIP ratio in the treatments; Control (C), Thiamin (Thi), Nitrogen (N), and Thiamin + Nitrogen (N_Thi) for each separate time point. Error bars show standard deviations for triplicates. For significant differences among treatments, see the text.


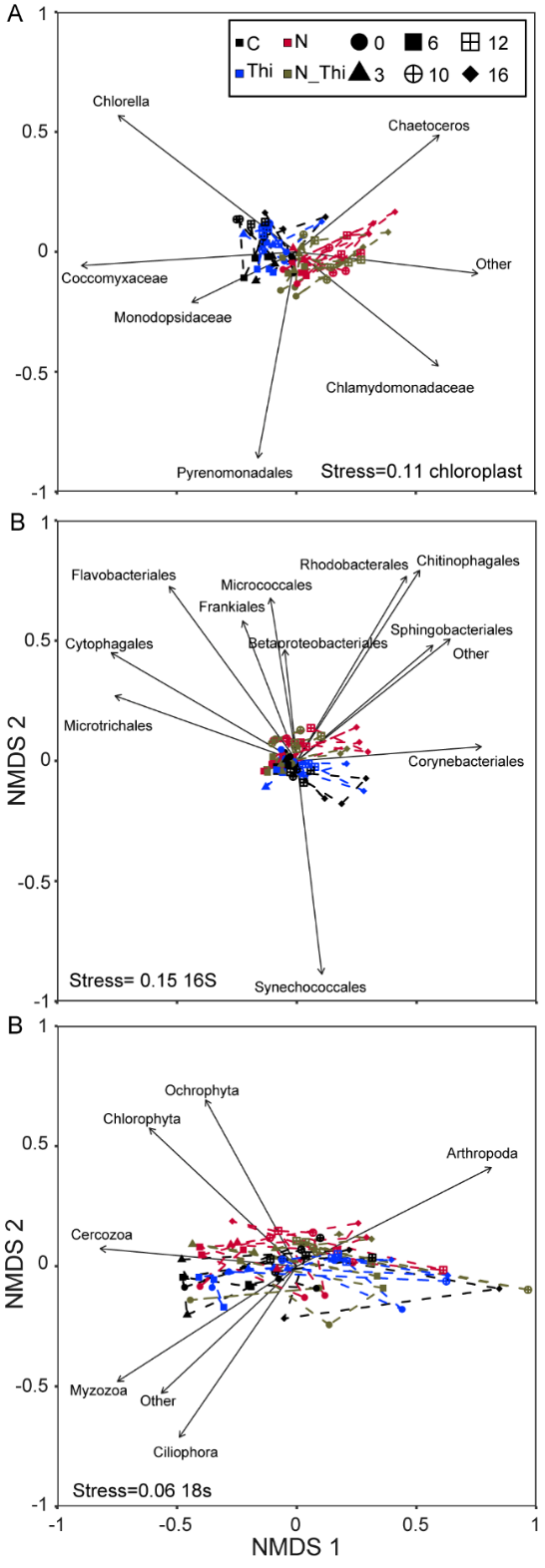


**S1 Figure S3.** Non-metric multidimensional scaling (NMDS) plot of community composition of 16S rRNA chloroplasts (A), 16S rRNA (B) and 18S rRNA (C). Each symbol represents median community composition (Bray-Curtis dissimilarity) for each replicate and time point (0, 3, 6, 10, 12, 16) whilst color represents treatment; Control (C), Thiamin (Thi), Nitrogen (N), and Thiamin + Nitrogen (N_Thi). Dashed lines connect replicates within a time point and treatment. Arrows illustrate fitted vectors.


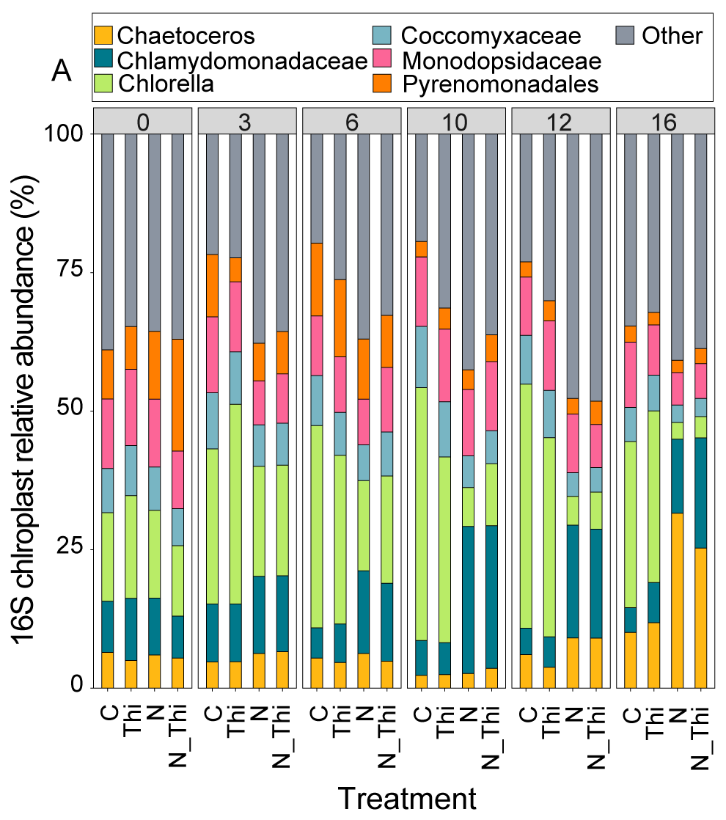


**S1 Figure S4**. Stacked bar graph of the average relative abundance and the taxonomical identification of the dominant chloroplast ASVs (>4% relative chloroplast read abundance) in the 16S rRNA gene amplicon libraries for each time point (Day 0, 3, 6, 10, 12 and 16) for triplicate treatments (C, Thi, N, and N_Thi). The remaining ASVs have been grouped in the category, Other, and ASVs with the same identification have been given indexes in the legend.


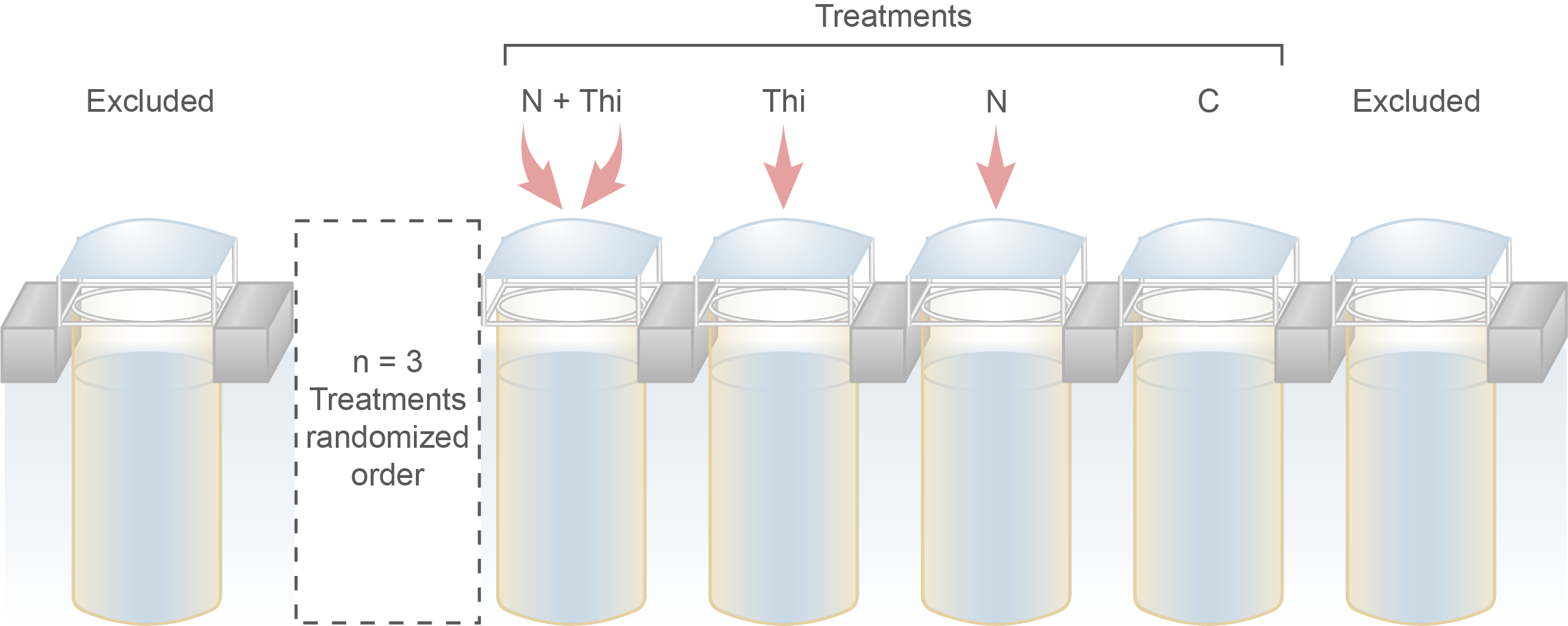


**S1 Figure S5**. The mesocosm study was performed by use of plastic bags (~1.9 m^3^) that were submerged in the sea at a coastal site in the Baltic Sea. Bags were in one row and each bag was covered by a Plexiglas sheet to avoid contamination from the atmosphere (e.g. birds defecating) and four treatments were applied with three replicates in each (C: low thiamin-low nitrogen; N: low thiamin- high nitrogen; Thi: high thiamin – low nitrogen and N+Thi: high thiamin – high nitrogen). The outermost mesocosm was not used to avoid any potential edge effects.

**S1 Table S1.** Number of high-quality reads (18S and 16S rRNA gene amplicons) and proportion of chloroplasts in 16S rRNA gene libraries per sample.

| **Treatment** | **Mesocosm** | **Day** | **18S reads** | **16S reads** | **chloroplast reads** | **% chloroplast reads** |
| --- | --- | --- | --- | --- | --- | --- |
| C | M1 | 0 | 75745 | 81391 | 4720 | 5% |
|  |  | 3 | 24753 | 10736 | 293 | 3% |
|  |  | 6 | 134625 | 35066 | 632 | 2% |
|  |  | 10 | 121581 | 54427 | 1933 | 3% |
|  |  | 12 | 150669 | 31930 | 807 | 2% |
|  |  | 16 | 236672 | 45545 | 1676 | 4% |
|  | M5 | 0 | 88028 | 48224 | 2747 | 5% |
|  |  | 3 | 137968 | 78952 | 3248 | 4% |
|  |  | 6 | 433975 | 36097 | 534 | 1% |
|  |  | 10 | 224895 | 54209 | 1634 | 3% |
|  |  | 12 | 446322 | 38574 | 1237 | 3% |
|  |  | 16 | 125238 | 19642 | 649 | 3% |
|  | M9 | 0 | 110651 | 46296 | 2398 | 5% |
|  |  | 3 | 115799 | 71829 | 2915 | 4% |
|  |  | 6 | 113232 | 70674 | 1980 | 3% |
|  |  | 10 | 219734 | 69624 | 1180 | 2% |
|  |  | 12 | 387485 | 57632 | 978 | 2% |
|  |  | 16 | 268079 | 69066 | 8863 | 11% |
| Thi | M3 | 0 | 100696 | 93774 | 5475 | 6% |
|  |  | 3 | 131872 | 40051 | 1133 | 3% |
|  |  | 6 | 118282 | 38895 | 740 | 2% |
|  |  | 10 | 19726 | 43215 | 1698 | 4% |
|  |  | 12 | 108006 | 45737 | 2078 | 4% |
|  |  | 16 | 265945 | 51052 | 1501 | 3% |
|  | M7 | 0 | 110532 | 48519 | 1915 | 4% |
|  |  | 3 | 103938 | 68391 | 2499 | 4% |
|  |  | 6 | 120475 | 34318 | 706 | 2% |
|  |  | 10 | 137309 | 92186 | 4629 | 5% |
|  |  | 12 | 341511 | 53041 | 2403 | 4% |
|  |  | 16 | 109091 | 100530 | 7106 | 7% |
|  | M11 | 0 | 37836 | 47554 | 2447 | 5% |
|  |  | 3 | 101640 | 13673 | 161 | 1% |
|  |  | 6 | 115527 | 77537 | 2377 | 3% |
|  |  | 10 | 160163 | 61451 | 1320 | 2% |
|  |  | 12 | 408731 | 46753 | 1119 | 2% |

Table S1 continued

| **Treatment** | **Mesocosm** | **Day** | **18S reads** | **16S reads** | **chloroplast reads** | **% chloroplast reads** |
| --- | --- | --- | --- | --- | --- | --- |
| N | M2 | 0 | 110737 | 59009 | 3958 | 6% |
|  |  | 3 | 137996 | 33575 | 2642 | 7% |
|  |  | 6 | 141618 | 32399 | 1238 | 4% |
|  |  | 10 | 138401 | 73952 | 3393 | 4% |
|  |  | 12 | 134385 | 26816 | 1250 | 4% |
|  |  | 16 | 261267 | 21133 | 2077 | 9% |
|  | M6 | 0 | 103511 | 47520 | 2354 | 5% |
|  |  | 3 | 86653 | 65816 | 6633 | 9% |
|  |  | 6 | 144081 | 39682 | 1181 | 3% |
|  |  | 10 | 201674 | 79055 | 4149 | 5% |
|  |  | 12 | 235303 | 50476 | 2814 | 5% |
|  |  | 16 | 145460 | 27213 | 1369 | 5% |
|  | M10 | 0 | 102089 | 46672 | 2660 | 5% |
|  |  | 3 | 122567 | 66936 | 4928 | 7% |
|  |  | 6 | 87527 | 60768 | 2866 | 5% |
|  |  | 10 | 180642 | 65414 | 1651 | 2% |
|  |  | 12 | 391599 | 69062 | 3354 | 5% |
|  |  | 16 | 370683 | 74751 | 7325 | 9% |
| NThi | M4 | 0 | 83132 | 43293 | 2338 | 5% |
|  |  | 3 | 88743 | 28137 | 1406 | 5% |
|  |  | 6 | 443259 | 37907 | 882 | 2% |
|  |  | 10 | 128296 | 44978 | 1042 | 2% |
|  |  | 12 | 442718 | 45820 | 2181 | 5% |
|  |  | 16 | 223529 | 53038 | 3401 | 6% |
|  | M8 | 0 | 90731 | 47988 | 3103 | 6% |
|  |  | 3 | 110670 | 66983 | 4137 | 6% |
|  |  | 6 | 42406 | 38463 | 1144 | 3% |
|  |  | 10 | 145053 | 76165 | 2210 | 3% |
|  |  | 12 | 321914 | 52544 | 3078 | 6% |
|  |  | 16 | 163559 | 23362 | 2364 | 9% |
|  | M12 | 0 | 79564 | 42871 | 2651 | 6% |
|  |  | 3 | 78957 | 37075 | 2627 | 7% |
|  |  | 6 | 108206 | 37630 | 816 | 2% |
|  |  | 10 | 46078 | 63340 | 2431 | 4% |
|  |  | 12 | 218716 | 98124 | 4740 | 5% |

**S1 Table S2**. Identification of individual zooplankton using microscopy and two sets of primers; the forward primer LCO-1490 in combination with HCO-Co-23585 (A) or HCO-21985 (B). na –poor PCR amplification. unknown –closest similarity match <80% similarity.

| Sample | Microscopy identification | Primer pair | Closest relative in GenBank | %similarity | Accession number |
| --- | --- | --- | --- | --- | --- |
| MicrothiPL_3 | Eurytemora | A | *Eurytemora affinis* | 98 | NC_046694 |
|  |  | B | *Eurytemora affinis* P4 | 99 | JQ822117 |
| MicrothiPL_4 | Eurytemora | A | *Eurytemora affinis* | 98 | NC_046694 |
|  |  | B | *Eurytemora affinis* ZISP:08SNM-334 | 100 | HM474015 |
| MicrothiPL_6 | Balanus | A | *Amphibalanus improvisus* frag6 | 100 | FJ845844 |
|  |  | B | *Amphibalanus improvisus* frag6 | 100 | FJ845844 |
| MicrothiPL_8 | Acartia | A | *Acartia bifilosa* MT02541 | 99 | JX995249 |
|  |  | B | *Acartia bifilosa* MT02541 | 99 | JX995249 |
| MicrothiPL_9 | Acartia | A | *Acartia bifilosa* MT02541 | 99 | JX995249 |
|  |  | B | *na* |  |  |
| MicrothiPL_12 | Podon | A | *na* |  |  |
|  |  | B | *na* |  |  |
| MicrothiPL_13 | Podon | A | *Acartia sp.* 10PROBE-18966 | 99 | MG314553 |
|  |  | B | *na* |  |  |
| MicrothiPL_14 | Podon | A | *unknown* |  |  |
|  |  | B | *na* |  |  |
| MicrothiPL_16 | Podon | A | *na* |  |  |
|  |  | B | *na* |  |  |
| MicrothiPL_19 | Evadne | A | *na* |  |  |
|  |  | B | *Mytilus trossulus* C 8843/C-A-37605 | 100 | KU925349 |
| MicrothiPL_20 | Evadne | A | *Evadne nordmanni* | 99 | AY075049 |
|  |  | B | *Eurytemora affinis* ZISP:08SNM-275 | 100 | HM473965 |
| MicrothiPL_21 | Evadne | A | *Synchaeta sp*. WM-2017b | 89 | LC215582 |
|  |  | B | *Synchaeta sp.* 16SK1Levico | 89 | JN936570 |
| MicrothiPL_22 | Temora | A | *Bermanella marisrubri* RED65 | 83 | CP051183 |
|  |  | B | *na* |  |  |
| MicrothiPL_23 | Temora | A | *na* |  |  |
|  |  | B | *Parvamoeba rugata* CCAP 1556/1 | 84 | JN202434 |
| MicrothiPL_29 | Cyclopoid | A | *Undinula vulgaris* | 82 | MN603005 |
|  |  | B | *Mesocyclops thermocyclopoides* MSOTH1 | 83 | KJ020572 |

**S2 Table S3**. Separate excel file.
